# Input-output functions of the nonlinear-distortion component of distortion-product otoacoustic emissions in normal and hearing-impaired human earsa)

**DOI:** 10.1121/1.4982923

**Published:** 2017-05-11

**Authors:** Dennis Zelle, Lisa Lorenz, John P. Thiericke, Anthony W. Gummer, Ernst Dalhoff

**Affiliations:** Section of Physiological Acoustics and Communication, Department of Otolaryngology, Eberhard-Karls-University Tübingen, Elfriede-Aulhorn-Straße 5, 72076 Tübingen, Germany

## Abstract

Distortion-product otoacoustic emissions (DPOAEs) arise in the cochlea in response to two tones with frequencies *f*_1_ and *f*_2_ and mainly consist of two components, a nonlinear-distortion and a coherent-reflection component. Wave interference between these components limits the accuracy of DPOAEs when evaluating the function of the cochlea with conventional continuous stimulus tones. Here, DPOAE components are separated in the time domain from DPOAE signals elicited with short stimulus pulses. The extracted nonlinear-distortion components are used to derive estimated distortion-product thresholds (EDPTs) from semi-logarithmic input-output (I/O) functions for 20 normal-hearing and 21 hearing-impaired subjects. I/O functions were measured with frequency-specific stimulus levels at eight frequencies *f*_2_ = 1,…, 8 kHz (*f*_2_/*f*_1_ = 1.2). For comparison, DPOAEs were also elicited with continuous primary tones. Both acquisition paradigms yielded EDPTs, which significantly correlated with behavioral thresholds (*p* < 0.001) and enabled derivation of estimated hearing thresholds (EHTs) from EDPTs using a linear regression relationship. DPOAE-component separation in the time domain significantly reduced the standard deviation of EHTs compared to that derived from continuous DPOAEs (*p* < 0.01). In conclusion, using frequency-specific stimulus levels and DPOAE-component separation increases the reliability of DPOAE I/O functions for assessing cochlear function and estimating behavioral thresholds.

## I. INTRODUCTION

The healthy cochlea amplifies sound by actively (Gold, 1948) enhancing vibrations of the basilar membrane at low to moderate sound pressure levels (Sellick *et al.*, 1982) and, thereby, establishes the high sensitivity, the large dynamic range, and the sharp tuning of the auditory system (for review, Robles and Ruggero, 2001). The system of biomechanical components involved in the amplification process is referred to as the cochlear amplifier, a term introduced in a review paper by Davis (1983). As a by-product of the amplification process, the cochlea emits sound waves measurable in the ear canal using a sensitive microphone, both in the absence of external sound, referred to as spontaneous otoacoustic emissions (SOAEs), and in response to external stimuli, referred to as evoked otoacoustic emissions (OAEs). OAEs are widely used in clinical routine as an objective and noninvasive measure of cochlear function, such as in newborns and young children or in serial monitoring of potentially ototoxic drugs (Probst *et al.*, 1991).

One type of OAE commonly used in clinical applications and research is the distortion product otoacoustic emission (DPOAE) which, by definition, is produced when stimulating simultaneously with two tones, with frequencies denoted by *f*_1_ and *f*_2_ where *f*_2_ > *f*_1_ (Kemp, 1979; Avan *et al.*, 2013). In humans, the most pronounced DPOAE is found at the cubic difference frequency *f*_DP_ = 2*f*_1_–*f*_2_ and is assumed to be comprised mainly of two components generated by different mechanisms at different sites along the basilar membrane (Brown *et al.*, 1996; Shera and Guinan, 1999). The first component arises directly from nonlinear interaction of the two traveling waves, which overlap maximally close to the tonotopic place of the *f*_2_ tone and simultaneously deflect the stereocilia of the outer hair cells (OHCs) with frequencies *f*_1_ and *f*_2_. Because of its nonlinear dependence on stereocilia deflection, the receptor current exhibits intermodulation products, which are coupled into the cochlear fluid as vibrations by mechanical forces from the electromechanical transducer of the OHC soma (Avan *et al.*, 2013). In the case of the cubic intermodulation product, the vibrations are evident as two traveling waves of frequency *f*_DP_. One of the waves propagates retrograde toward the stapes and is referred to as the nonlinear-distortion component, in consequence of its direct origin in nonlinearity. The other wave propagates anterograde to the tonotopic place of *f*_DP_, where coherent reflection, presumably due to irregularities of mechanical properties along the cochlea, gives rise to another DPOAE component (Shera and Guinan, 1999), referred to as the coherent-reflection component.

DPOAE amplitudes or levels are known to decrease with increasing hearing thresholds (Probst and Hauser, 1990; Gorga *et al.*, 1993), an observation which is exploited for diagnostic purposes. However, the high variability of DPOAE amplitudes across subjects (Probst *et al.*, 1991) and insufficient performance at low frequencies (Gorga *et al.*, 1993) limit their accuracy for assessing behavioral thresholds. An alternative approach utilizes the dependence of DPOAE level on the stimulus level, L_2_, of the second stimulus tone, called the DPOAE input-output (I/O) function, to obtain DPOAEs at low stimulus levels. This approach has been shown to enhance the sensitivity of DPOAEs for detecting cochlear damage and to increase their correlation with auditory thresholds (Gaskill and Brown, 1990; Kummer *et al.*, 1998; Dorn *et al.*, 2001). Boege and Janssen (2002) introduced a refined procedure based on a semi-logarithmic plot of the DPOAE I/O function. Using stimulus levels chosen according to the so-called scissor paradigm, L_1_ = 0.4L_2_ + 39 dB (Kummer *et al.*, 1998), the DPOAE-pressure amplitude was found to depend linearly on L_2_. This linearized form of the DPOAE I/O-function enabled determination of the so-called estimated distortion product threshold (EDPT) by extrapolating the linear regression line to the abscissa (Boege and Janssen, 2002). The EDPTs were shown to correlate significantly with auditory thresholds (Boege and Janssen, 2002; Gorga *et al.*, 2003; Neely *et al.*, 2009). However, despite this linearization of the DPOAE I/O functions, the standard deviation of the differences between auditory thresholds and EDPTs was higher than 10 dB, with individual threshold estimation errors being as much as 30 dB or more (Schmuziger *et al.*, 2006).

One reason for the limited test performance when using DPOAEs to detect hearing loss or relating DPOAE thresholds to behavioral thresholds is interference between the nonlinear-distortion component and the coherent-reflection component, each of which has frequency *f*_DP_. When stimulating the cochlea with conventional continuous primary tones, wave interference occurs because the DPOAE signal measured in the ear canal is the vector sum of these two signal components (Brown *et al.*, 1996), which is then usually quantified by spectral analysis (Shera and Guinan, 1999; Avan *et al.*, 2013). In contrast to the above-mentioned studies, the present work investigates the impact of interference between the DPOAE components when using the semi-logarithmic DPOAE I/O functions to estimate behavioral thresholds.

While the nonlinear-distortion component exhibits relatively constant phase as function of *f*_2_, the phase of the coherent-reflection component changes considerably with *f*_2_ (Shera and Guinan, 1999). This frequency-dependent phase difference between the two components leads to quasi-periodic variation of DPOAE amplitude as function of *f*_2_, commonly referred to as DPOAE fine structure (Gaskill and Brown, 1990; Heitmann *et al.*, 1998; Talmadge *et al.*, 1999), and is characterized by amplitude maxima and minima corresponding to constructive and destructive interference, respectively. Depending on the relative differences in amplitude and phase between the two components, the measured DPOAE response might not accurately reflect the functional state of the cochlea at the *f*_2_ place. For example, the two DPOAE components might almost completely cancel when the phase difference is close to 180° and their amplitudes are similar. Moreover, the locations of minima and maxima of the DPOAE fine structure can shift in frequency with increasing stimulus levels (He and Schmiedt, 1993). Such frequency shifts become apparent as valleys and peaks in three-dimensional plots of DPOAE amplitude as function of L_1_ and L_2_ (Zelle *et al.*, 2015a). These intensity-dependent interference effects can cause considerable deformations in DPOAE I/O functions, yielding large standard deviations in the estimates of slope and EDPT (Mauermann and Kollmeier, 2004; Dalhoff *et al.*, 2013).

The two DPOAE components become distinguishable as short- and long latency components when converting a DP-gram into its temporal counterpart using an inverse fast Fourier transform (IFFT) (Stover *et al.*, 1996). The IFFT technique can be applied to reduce fine structure by exploiting the shorter latency of the nonlinear-distortion component relative to the coherent-reflection component in the time domain (Kalluri and Shera, 2001; Mauermann and Kollmeier, 2004). Similarly, acquisition paradigms with swept primary tones utilize the different latencies to estimate the nonlinear-distortion component using a least-squares-fit (LSF) algorithm (Long *et al.*, 2008; Abdala *et al.*, 2015) or by means of time-frequency filtering (Moleti *et al.*, 2012). Despite offering reliable extraction of the nonlinear-distortion component, these techniques either rely on time-consuming recordings of DP-grams or employ chirps with high frequency resolution at the expense of acquisition time, which can be disadvantageous if I/O functions at only a few frequencies are of interest, as in a clinical setting. An alternative method to obtain DPOAEs solely expressing the functional state of the cochlea at the *f*_2_-tonotopic place, is the use of a third tone to suppress the coherent-reflection component (Heitmann *et al.*, 1998). This technique does not require recordings at multiple frequencies, but fails to improve accuracy or reliability when assessing hearing status (Dhar and Shaffer, 2004; Johnson *et al.*, 2006b; Johnson *et al.*, 2007).

The presence of two DPOAE components also becomes evident during the onset and the offset of the DPOAE signal, when using a pulsed *f*_2_ stimulus and analyzing the DPOAE signal in the time domain (Whitehead *et al.*, 1996; Talmadge *et al.*, 1999; Konrad-Martin and Keefe, 2005). Because of their different latencies, the nonlinear-distortion component can be separated from the coherent-reflection component by a method called onset decomposition (OD) (Vetešník *et al.*, 2009). This technique samples the envelope of the DPOAE signal at a time instant before the coherent-reflection component starts to interfere. Although a very promising technique, OD as was implemented by Vetešník *et al.* (2009) was unnecessarily time-consuming because the stimulus pulse duration was longer than required as the signal information after the sampling instant was discarded.

The present study extends previous research by using the OD technique to extract the nonlinear-distortion component from the DPOAE signal produced by stimuli of short duration and then using the DPOAE I/O function of the nonlinear-distortion component to deliver the EDPT and estimate auditory threshold. These so-called short-pulse DPOAEs utilize brief *f*_2_ pulses with a duration similar to the relative delay between the two DPOAE components, in order to facilitate component separation in the time domain (Zelle *et al.*, 2013). In this way, semi-logarithmic I/O functions based on the nonlinear-distortion component allow estimation of auditory thresholds without artifacts due to interference of the two DPOAE components. Moreover, DPOAE recordings were made with optimized, frequency-dependent stimulus levels (Zelle *et al.*, 2015a), which account for the different compression of the primary-tone traveling waves at the generation site of the DPOAE close to the *f*_2_-tonotopic place (Robles and Ruggero, 2001). In contrast to previously reported primary-tone levels (Kummer *et al.*, 1998; Johnson *et al.*, 2006a), the optimal stimulus-intensity functions used here were based solely on the nonlinear-distortion component. For comparison, DPOAE I/O functions were also acquired conventionally with continuous primary-tone stimulation. Experiments were conducted with normal-hearing and hearing-impaired subjects in a clinically relevant frequency range from 1 to 8 kHz. Estimates of auditory thresholds based on both short-pulse and continuous DPOAEs are compared to behavioral thresholds measured by Békésy audiometry to evaluate the utility of short-pulse DPOAEs for objectively determining behavioral thresholds. It is shown that the short-pulse stimulus and analysis paradigms allow estimation of auditory threshold with hitherto unprecedented high accuracy.

## II. MATERIALS AND METHODS

### A. Study design and subjects

DPOAE I/O functions were recorded unilaterally from 20 normal-hearing and 21 hearing-impaired subjects with sensorineural hearing loss. Subjects were between 18 and 70 years and the normal-hearing group was significantly younger (mean age: 27.6 ± 4.2 years) compared to the hearing-loss subjects (mean age: 49.7 ± 13.0 years, *p* < 0.001). In order to identify hearing-impaired ears, behavioral thresholds (BTs) were recorded with clinical pure-tone audiometry (Audiometer AT 900, Auritec, Medizindiagnostische Systeme, Hamburg, Germany). Subjects were classified as normal-hearing if all BTs for frequencies between 1 and 8 kHz were better than 20 dB hearing level (HL). BTs for hearing-impaired ears ranged from 0 to 77 dB HL with an average value of 24 ± 18 dB HL (normal-hearing: 7 ± 5 dB HL). All subjects were free of any conductive hearing impairment as ascertained by standard 226-Hz tympanometry (Madsen-Zodiac 901, GN Otometrics, Münster, Germany) and otoscopy. Measurements of clinical, notched-noise, auditory-brainstem responses (ABR) for 1, 2, and 4 kHz and stimulus levels from 25 to 75 dB nHL in 10-dB steps (Evoselect ERA system, Pilot Blankenfelde Medizinisch-Elektronische Geräte, Blankenfelde, Germany) and acoustic reflex measurements at 0.5, 1, 2, and 4 kHz (Madsen-Zodiac 901) were used to exclude possible severe neural conditions for the hearing-impaired group. Subjects were included only if ABR waves V were detectable for at least one of the investigated frequencies. To avoid false-positive exclusions in cases without identifiable ABR signals, subjects were also included if at least one ipsilateral stapedius reflex could be detected. In 17 hearing-impaired subjects, ABR-wave V was detectable for at least one test frequency for stimulus levels equal to or below 65 dB nHL. In the remaining four subjects, ipsilateral stapedius reflexes were detectable at two or more frequencies. The subjects had no history of tinnitus.

The study was approved by the Ethics Committee of the University of Tübingen in accordance with the Declaration of Helsinki for human experiments. An informed consent in written form was provided by all subjects.

### B. Measurement system and calibration

OAE measurements and Békésy audiometry were performed unilaterally using an ER-10 C DPOAE probe-microphone system (Etymotic Research, Elk Grove Village, IL) connected to a 16-bit analog output card and a 24-bit signal acquisition card (NI PCI 6733 and NI PCI 4472, National Instruments, Austin, TX) situated in a commercially available PC. The sampling frequency was 102.4 kHz. Stimulus generation and data acquisition were controlled by a custom-built toolbox implemented in LabVIEW (version 12.0, National Instruments, Austin, TX). The sound pressure of the ER-10 C speakers was ascertained by in-ear calibration, which was repeated every 120 to 240 s depending on the acquisition progress. Both the output of the speakers and the recorded microphone signal were corrected for the transfer functions of an artificial ear simulator (B&K type 4157, Brüel & Kjær, Nærum, Denmark) and of the ER-10 C microphone to yield DPOAEs, which are considered to correspond to recordings close to the tympanic membrane. Further details of the calibration routine are given elsewhere (Zelle *et al.*, 2015a). Signal post-processing and data analysis were done in matlab (version 9.0, MathWorks, Natick, MA).

### C. Assessment of behavioral thresholds

A modified method of Békésy tracking audiometry was performed using the ER-10 C ear probe to assess behavioral thresholds in each subject directly before the OAE data acquisition started. The sound pressure of the continuous tone was controlled by the data acquisition software while the subject was required to indicate perception of the stimulus by pressing or releasing a button. The output level, L, started at −20 dB sound pressure level (SPL), well below hearing threshold, and increased in 0.1-dB steps with an alteration rate of 8 dB/s. The acquisition setup gradually decreased the intensity-rate change to avoid clicks in the presentation of tones with high output level (ultimately, 2 dB/s at L > 60 dB SPL). The subject was instructed to press and hold down a button if the sound was perceived, thereby establishing an upper pure-tone threshold. While the button was held down, the system decreased the output level until the subject lost perception of the sound. Releasing the button indicated a lower pure-tone threshold. The mean value of the lower and upper threshold provided an estimate of the auditory threshold. On average, the elapsed time between the detection of these two thresholds was 2.25 ± 0.79 s. The maximum output level was set to 85 dB SPL. As in Dalhoff *et al.* (2013), behavioral thresholds were recorded not only at each *f*_2_ frequency, but additionally at five to nine (mostly seven) neighboring frequencies, in order to account for the frequency-dependent bandwidth of the short-pulse *f*_2_ stimulus. The frequency range spanned by the lowest and highest neighboring frequencies was 80 Hz at *f*_2_ = 1 kHz and increased to 480 Hz at *f*_2_ = 8 kHz. These frequency ranges were similar to the bandwidths of the associated *f*_2_ pulses.

Three successive Békésy measurements were recorded and averaged to obtain a reliable estimate of the behavioral threshold. To reduce the impact of outliers, a correction algorithm similar to the one introduced in Dalhoff *et al.* (2013) was implemented. For each frequency group formed by *f*_2_ and its neighboring frequencies, the median values and the standard deviations were computed separately for the lower and upper thresholds across the three Békésy recordings. A behavioral threshold which differed from a lower- or upper-threshold median by more than three times the associated standard deviation was classified as an outlier and replaced by the median value of the lower or upper threshold of the frequency group. This procedure enabled frequency-specific outlier correction even for hearing-impaired subjects. Finally, the estimates of the behavioral thresholds at the *f*_2_ frequencies, denoted as L_BT_, were computed by averaging across frequencies for each frequency group, in order to mimic the spectral spread of the pulsed DPOAE stimuli.

### D. DPOAE acquisition and analysis

**FIG. 1.**
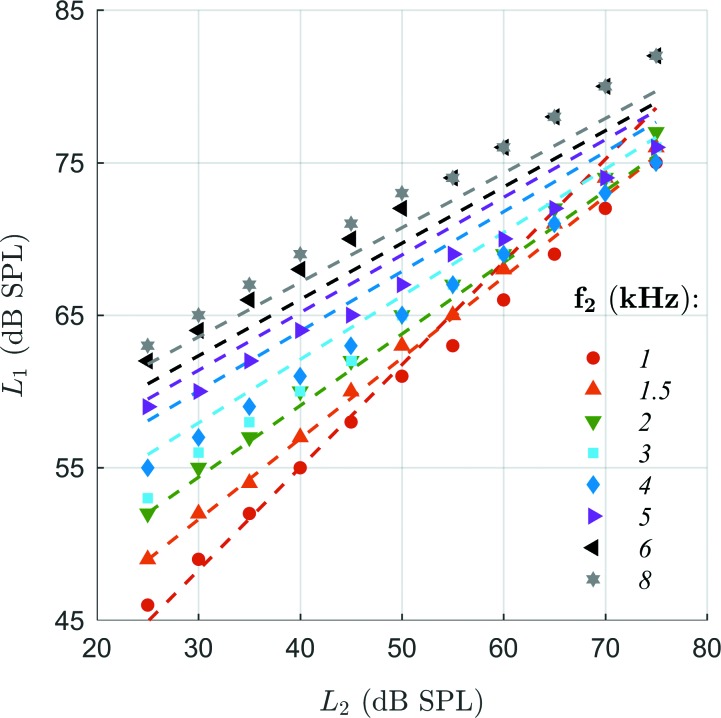
Frequency-specific stimulus level pairs accounting for compressibility of the basilar-membrane response at the *f*_2_-tonotopic place, optimized to facilitate DPOAE acquisition with maximum amplitude of the nonlinear-distortion component. Dashed lines show the optimal stimulus levels of the *f*_1_ tones, L_1_, as a function of the stimulus levels of the *f*_2_ tones, L_2_, provided by the empirical relation in Zelle *et al.* [2015a; their Eq. (5)] which was derived experimentally from normal-hearing adult subjects. Symbols are stimulus-level pairs used here; they derive from preliminary results of that study. The frequencies *f*_2_ were chosen to correspond to frequencies commonly used in clinical routine.

DPOAE I/O functions were collected at eight frequencies for 1 ≤ *f*_2_ ≤ 8 kHz with a constant frequency ratio of *f*_2_/*f*_1_ = 1.2. L_2_ values ranged from 25 to 75 dB SPL in 5-dB steps with frequency-dependent L_1_ values representing preliminary results based on a subset of a recently published study (Zelle *et al.*, 2015a). That study proposed optimized stimulus level pairs, which maximize the amplitude of the nonlinear-distortion component. Figure 1 shows the frequency-specific levels of the *f*_1_ tone, L_1_, as a function of L_2_ according to

$$L_{1}\left(f_{2},L_{2}\right)=a\left(f_{2}\right)L_{2}+b\left(f_{2}\right),$$ (1)

from Zelle *et al.* (2015a) (dashed lines) with the frequency-dependence of a and b given by their Eq. (5). The average deviation of the stimulus level pairs used here (symbols) from the optimal stimulus-level path was 0.30 ± 1.87 dB, which is within the standard deviation of the population data in Zelle *et al.* (2015a).

#### 1. Pulse stimulation

DPOAEs were evoked using a recently introduced *multi-frequency acquisition* paradigm, which utilizes a sequence of short stimulus pulses for a given set of primary-tone levels L_1_ and L_2_, to enable extraction of the nonlinear-distortion component for multiple stimulus-frequency pairs with frequencies *f*_1,i_ and *f*_2,i_ from a single recording (Zelle *et al.*, 2014; Zelle *et al.*, 2015a). Each sequence was composed of four stimulus pairs, i = 1,…, 4, each of which comprised a *f*_1,i_ pulse of 30-ms duration and a *f*_2,i_ pulse with frequency-dependent half width corresponding to the expected relative delay between the two DPOAE components, estimated from the results of Vetešník *et al.* (2009). The sequence of the frequency pairs was chosen to provide sufficient distance in both the frequency and the time domain to enable unambiguous extraction of the DPOAE signal by band-pass filtering. For each L_2_ value, two separate measurements were performed with different frequency sequences of either *f*_2_ = [1, 3, 1.5, 6] or *f*_2_ = [8, 4, 2, 5] kHz, yielding a total duration of a single acquisition block of 120 ms. A detailed description of the acquisition technique can be found elsewhere (Zelle *et al.*, 2015a). Cancellation of the stimulus pulses and related stimulus-frequency OAEs was achieved by suitable phase shifts in four consecutive acquisition blocks together with ensemble averaging (Whitehead *et al.*, 1996). Signal averaging was performed until the DPOAE associated with the lowest signal-to-noise ratio (SNR) in the sequence, typically at *f*_2_ = 1 or 8 kHz, yielded a SNR of at least 10 dB, called the 10-dB SNR criterion, or a maximum number of 400 acquisition blocks was reached. Acquisition blocks not enhancing the SNR for a specific DPOAE were excluded from averaging.

For each stimulus pair, the corresponding DPOAE signal, $p_{i}\left(t\right)$, at the frequency *f*_DP,i_ = 2*f*_1,i_ − *f*_2,i_, was extracted from the averaged datasets by zero-phase band-pass filtering using a finite impulse response (FIR) filter with an order of 1200 and filter coefficients computed using a Hamming window. The filter bandwidths were defined as

$$B\left({\tilde{f}}_{2},{\tilde{L}}_{2}\right)=2f_{c}\left({\tilde{f}}_{2},{\tilde{L}}_{2}\right),$$ (2)

with the cutoff frequency $f_{c}$ defined as the frequency at which the attenuation of the filter was 6 dB, the normalized frequency ${\tilde{f}}_{2}=f_{2}/f_{2,max}$, and the normalized level ${\tilde{L}}_{2}=L_{2}/L_{2,max}$ of the corresponding *f*_2_ stimulus. The maximum values were $f_{2,max}$ = 8 kHz and $L_{2,max}$ = 75 dB SPL. If a DPOAE signal did not comply with the 10-dB SNR criterion, the bandwidth was gradually reduced using an iterative algorithm and an initial bandwidth defined as

$$B_{0}\left({\tilde{f}}_{2},{\tilde{L}}_{2}\right)=\left(c_{1}{\tilde{L}}_{2}+c_{2}\right)e^{\left(c_{3}/{\tilde{f}}_{2}\right)},$$ (3)

with the parameters $c_{1}$ = 0.49 Hz, $c_{2}$ = 0.71 Hz, and the dimensionless parameter $c_{3}$ = −0.245. These parameters were determined by applying a nonlinear least-squares curve fitting method to data from the normal-hearing subset. The iterative algorithm decreased the bandwidth with a scaling parameter α according to

$$B_{k+1}\left({\tilde{f}}_{2},{\tilde{L}}_{2}\right)=\alpha B_{k}\left({\tilde{f}}_{2},{\tilde{L}}_{2}\right),$$ (4)

with α = 0.9, until the 10-dB SNR criterion was satisfied or a maximum of ten iterations was reached.

The SNR for the short-pulse DPOAE measurements was defined by the ratio of the amplitude of the extracted nonlinear-distortion component, ${\hat{P}}_{OD}$, and a noise estimate in the time domain computed as the root-mean-square value of remaining signal parts without DPOAE components or other coherent signals. This iterative adaptation of the filter bandwidth increased the detection rate of DPOAEs in subjects with generally low SNR or in hearing-impaired subjects, while reducing the filter effect on DPOAE pulse responses with large amplitudes. Due to the broadband character of the pulsed signals, narrowing the bandwidth reduced the DPOAE amplitude and, therefore, limited the potential improvement of SNR by means of band-pass filtering.

#### 2. Continuous stimulation

For comparison with conventional acquisition paradigms, DPOAEs were also recorded with continuous primary tones and the DPOAE amplitude was evaluated in the frequency domain by sampling the amplitude of the spectrum at the frequency bin associated with *f*_DP_. This yielded DPOAEs which represent the vector sum of the nonlinear-distortion component and the coherent-reflection component (Brown *et al.*, 1996). The frequencies of both stimulus tones were adjusted to yield an integer number of periods within the acquisition-block length of 100 ms. This adjustment resulted in a slight deviation (magnitude ≤0.0048) from the constant frequency ratio of 1.2 for some stimulus pairs. Data acquisition was continued until a SNR of at least 10 dB or a maximum iteration number of 100 was reached. Zero-phase high-pass filtering using a FIR filter with a filter order of 1024 was applied to each acquisition block before ensemble averaging. Filter coefficients were computed using a Hamming window with a 3-dB cutoff frequency of 290 Hz, which yielded sufficient attenuation of unwanted low-frequency signals (at least 50 dB below 80 Hz). Because of high-pass filtering, windowing was not required before computing the amplitude spectrum using the fast Fourier transform. Again, acquisition blocks which did not improve the SNR were not included in the ensemble averaging.

#### 3. Extraction of nonlinear-distortion product components

**FIG. 2.**
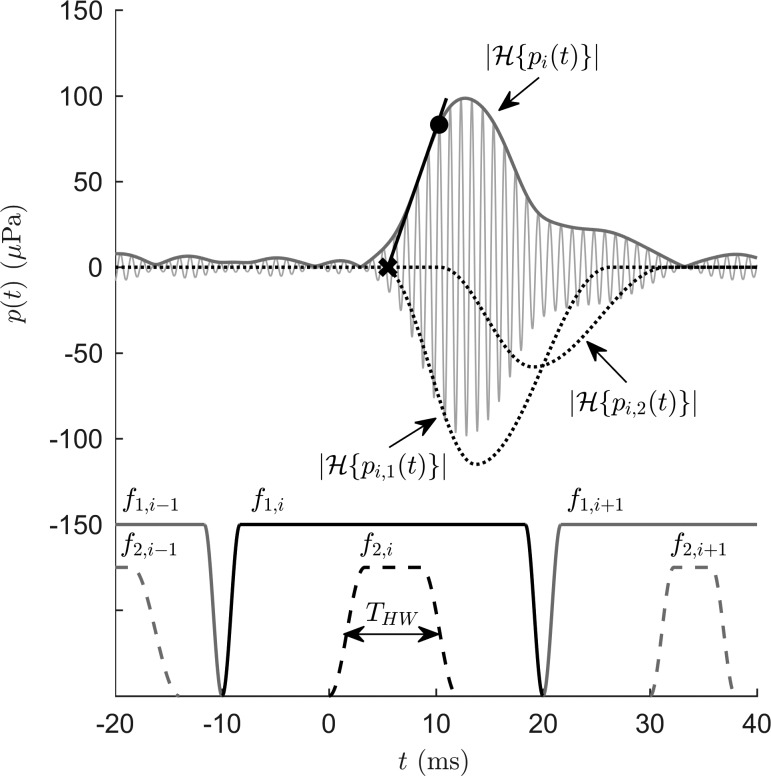
Stimulus and analysis paradigms. Upper part: Short-pulse DPOAE signal, $p_{i}\left(t\right)$, corresponding to the stimulus-frequency pair $f_{1,i}$ and $f_{2,i}$ after ensemble averaging and band-pass filtering (thin gray line), and its envelope computed as the absolute value of its Hilbert transform, $\left|H\left\{p_{i}\left(t\right)\right\}\right|$ (thick dark-gray line). Black dotted lines indicate envelopes of the nonlinear-distortion component, $\left|H\left\{p_{i,1}\left(t\right)\right\}\right|$, and the coherent-reflection component, $\left|H\left\{p_{i,2}\left(t\right)\right\}\right|$, extracted from the DPOAE signal using a nonlinear least-square curve fitting algorithm (Zelle *et al.*, 2013; Zelle *et al.*, 2015b). For visualization purposes, $\left|H\left\{p_{i,1}\left(t\right)\right\}\right|$ and $\left|H\left\{p_{i,2}\left(t\right)\right\}\right|$ are shown in reverse *y*-direction. The shorter latency of $p_{i,1}\left(t\right)$ enables separation of the two DPOAE components in the time domain. An automated detection algorithm computes the onset of the DPOAE signal (black cross) as the intersection of the tangent (black line) with the abscissa. Using this DPOAE onset, the sampling instant for onset decomposition (black dot) is chosen according to Eq. (5) to estimate the amplitude of $p_{i,1}\left(t\right)$ before $p_{i,2}\left(t\right)$ starts to interfere. Lower part: Schematic of the arrangement of the stimulus pairs (amplitudes not to scale) interlaced in the time domain with *f*_1_ pulses of 30-ms duration and *f*_2_ pulses of frequency-dependent half widths, T_HW_. Data from subject S054, *f*_2_ = 1.5 kHz, L_2_ = 45 dB SPL.

For the short-pulse DPOAE data, the nonlinear-distortion component was extracted in the time domain from the averaged and filtered dataset using an adapted version of the onset-decomposition technique introduced by Vetešník *et al.* (2009). This method samples the envelope of the DPOAE signal to obtain an estimate of the amplitude of the nonlinear-distortion component ${\hat{P}}_{OD}$ (black dot in Fig. 2) at a time point before interference with the coherent-reflection component begins. The envelope was obtained from the absolute value of the Hilbert transform of the DPOAE signal, $\left|H\left\{p_{i}\left(t\right)\right\}\right|$.

In order to achieve reliable separation of the two DPOAE components, the OD method requires *a priori* knowledge of DPOAE latencies for proper selection of the sampling instant. However, latencies of OAEs vary across subjects, depend on stimulus frequency and level (Stover *et al.*, 1996; Zelle *et al.*, 2015b), and are expected to change with hearing status (Engdahl and Kemp, 1996; Konrad-Martin and Keefe, 2005). Therefore, the OD technique was extended with an automated signal-detection algorithm to determine the sampling instant independently of the individual DPOAE latency. This algorithm detects the local maximum $\hat{P}$ of $p_{i}\left(t\right)$ closest to the onset of the *f*_2,i_ pulse, T_2,i_, and sets a tangent (black line, Fig. 2) at the inflection point that is located nearest to $\hat{P}$ and which exhibits a curvature change from convex to concave. The intersection point of the tangent with the abscissa yields an estimate of the DPOAE onset T_0_ (black cross, Fig. 2). Then, the sampling instant for OD is computed by

$$T_{OD}=T_{0}+\frac{2}{3}\left(T_{\hat{P}}-T_{0}\right),$$ (5)

where $T_{\hat{P}}$ is the time instant of the local maximum $\hat{P}$ and the factor $\frac{2}{3}$ was chosen empirically to avoid estimation errors due to a constructively interfering coherent-reflection component.

**FIG. 3.**
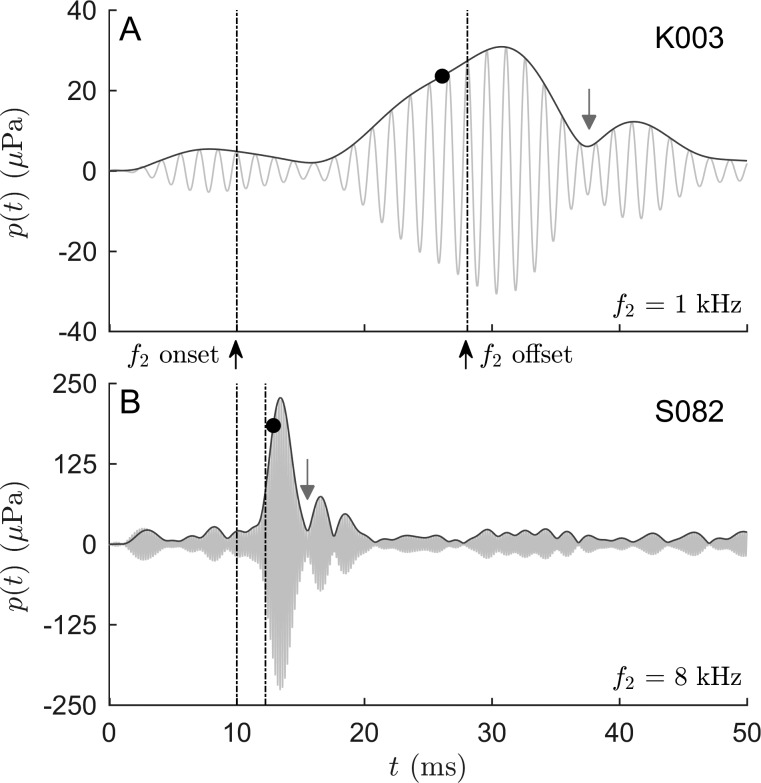
Short-pulse DPOAE responses (gray lines) recorded at *f*_2_ = 1 kHz (A) and 8 kHz (B) with L_2_ = 45 dB SPL. Dark-gray lines depict the envelope of the DPOAE with the black dots indicating the amplitudes of the nonlinear-distortion component estimated by onset decomposition, ${\hat{P}}_{OD}$. Dash-dotted lines represent the onset and offset of the *f*_2_ pulses with frequency-specific full widths at half maximum corresponding to the expected delay between the two DPOAE components. With increasing stimulus frequency, both latency and duration of the DPOAE responses decrease considerably. The automated signal-detection algorithm accounts for individual variations in the delay to enable reliable, objective DPOAE-component separation using OD. In both examples, a notch in the DPOAE signal (gray arrow) indicates the presence of the two DPOAE components. For subject K003 (A), the notch is associated with a phase jump of 152° in the instantaneous phase (not shown) suggesting destructive interference. For subject S082 (B), the associated phase jump is 321°, which indicates that the notch stems from a delay between the two DPOAE components exceeding the duration of the nonlinear-distortion component.

Figure 3 shows two short-pulse DPOAE responses recorded at 1 and 8 kHz, where both DPOAE components are evident from a notch in the time response (for details, see figure caption). Despite the onset of the *f*_2_ primary being identical in both examples, the delays of the DPOAE responses are considerably different. Using this automated signal-detection algorithm, the OD-technique was able to estimate the amplitude of the nonlinear-distortion component [black dot in Figs. 3(A) and 3(B)] before wave interference began, regardless of DPOAE latency.

### E. Determination of estimated distortion-product thresholds

Semi-logarithmic DPOAE I/O functions were derived from the amplitudes of the extracted nonlinear-distortion components for short-pulse stimulation and from the amplitude spectra of the DPOAE signals for continuous stimulation. For each *f*_2_, the I/O function was linearly extrapolated to the abscissa to yield the EDPT, by definition, the L_2_ value at which the DPOAE amplitude is equal to zero (Boege and Janssen, 2002; Gorga *et al.*, 2003). Only DPOAEs complying with the 10-dB SNR criterion (Sec. II D) were included in the regression analysis. At least three data points were required for the regression analysis, otherwise the I/O function was excluded from the data set. EDPTs were accepted for auditory-threshold estimation if they complied with the three objective evaluation criteria introduced in Boege and Janssen (2002): (1) a squared correlation coefficient of $r_{I/O}^{2}$ ≥ 0.8, (2) a standard deviation of the EDPT of $\sigma_{EDPT}$ ≤ 10 dB, and (3) a slope of the regression line of $s_{I/O}$ ≥ 0.2 μPa/dB SPL. Furthermore, EDPTs smaller than −10 dB SPL were excluded from further analysis because this criterion was shown to improve the performance of auditory-threshold prediction by preventing the inclusion of physiologically unrealistic, low EDPTs (Gorga *et al.*, 2003; Dalhoff *et al.*, 2013).

Approximately 38% of the semi-logarithmic I/O functions acquired with continuous primary tones and 25% of the semi-logarithmic I/O functions recorded with short-pulse stimulation exhibited extensive deviation from the expected straight-line behavior, especially at high stimulus levels where saturation was observed. Some I/O functions also showed “deformations” (e.g., notches), particularly at moderate levels, which were evident for both continuous and short-pulse stimulation. Therefore, a correction algorithm was implemented, similar to the *saturation-correction* algorithm introduced by Dalhoff *et al.* (2013), to increase the accuracy of the linear regression analysis at low-to-moderate levels.

Beginning at the highest stimulus level, the correction algorithm used an automated procedure to remove a set of sequential data points if they deviated from the presumed linear relationship normally apparent at low-to-moderate levels. The algorithm of Dalhoff *et al.* (2013) was extended by using not only $r_{I/O}^{2}$ but all three statistical evaluation parameters to find a suitable set of data points for regression analysis. For a given *f*_2_, let N be the number of stimulus levels for which the 10-dB SNR criterion was satisfied (Sec. II D) and M the maximum number of stimulus levels (N ≤ M). The levels associated with an I/O function are numbered sequentially from L_2,1_ at the lowest level to L_2,M_ at the highest level. Since an I/O function requires at least three valid data points, the removal of high-level data points allows N – 2 possible solutions. Each solution is identified by an integer j representing the number of data points removed from the I/O function; that is, j ranges from 0 to N – 3. Then, N – 2 candidate vectors comprising the three statistical evaluation parameters were defined as $z_{j}={\left[\begin{array}{ccc} r_{I/O,j}^{2} & s_{I/O,j} & \sigma_{EDPT,j} \end{array}\;\right]}^{T}$, where the superscript ^*T*^ denotes the transpose. In order to select the highest value of L_2_ to be included in the regression analysis, for each candidate vector the Euclidean norm $\xi_{j}$ was computed according to

$$\xi_{j}=\left\|\frac{z_{j}-z_{utopia}}{z_{nadir}-z_{utopia}}\right\|,$$ (6)

where $z_{\mathrm{nadir}}={\left[\begin{array}{ccc} \min\left\{r_{I/O}^{2}\right\} & \min\left\{s_{I/O}\right\} & \max\left\{\sigma_{EDPT}\right\} \end{array}\right]}^{T}\;$ is the vector of the worst-case evaluation parameters and $z_{\mathrm{utopia}}={\left[\begin{array}{ccc} \max\left\{r_{I/O}^{2}\right\} & \max\left\{s_{I/O}\right\} & \min\left\{\sigma_{EDPT}\right\} \end{array}\right]}^{T}$ is the vector of the best possible, but generally unachievable combination of evaluation parameters. Hereby, for a given evaluation parameter, { } denotes the set of those parameters for j = 0,…, N – 3. Both vectors were determined from the (N – 2)-tuple of possible I/O functions. $\xi_{j}$ can take values from 0 to $\sqrt{3}$ with a value approaching 0 representing the best possible solution. Finally, the value j associated with the minimum of $\xi_{j}$, denoted by j_min_, was used to determine the index k = M – j_min_ of the largest stimulus level, L_2,k_, to be included in the regression analysis. As examples, j_min_ = 0 represents the unaltered I/O function where all available DPOAE amplitudes will be included in the computation of the regression line, while j_min_ = 8 indicates the exclusion of DPOAE amplitudes associated with the eight highest L_2_ values, resulting in L_2,k=3_ = 35 dB SPL. This method not only corrects for saturation effects, but also accounts for deviations from a straight-line semi-logarithmic I/O function induced by deviations from the optimal stimulus-level path [Eq. (1)] or by two-component interference. Therefore, the algorithm is referred to as the *high-level correction* algorithm, abbreviated as the HLC algorithm.

### F. Estimation of fine-structure contribution

To estimate the number of I/O functions affected by two-component interference, the nonlinear-distortion component *and* the coherent-reflection component were extracted from the short-pulse DPOAE signal elicited at L_2_ = 45 dB SPL. In the case of insufficient SNR at L_2_ = 45 dB SPL, the DPOAE signal at the first higher L_2_ complying with the 10-dB SNR criterion was selected for the analysis. Extraction was achieved by decomposing the DPOAE signal into so-called *pulse basis functions* (PBFs) (Zelle *et al.*, 2013). PBF decomposition assumes that the short-pulse DPOAE signal can be described by a vector sum of windowed sine waves, called the pulse basis functions. The sum is least-mean-square fitted to the recorded signal in the time domain to extract the underlying DPOAE components. The fitted function was accepted for further analysis if the normalized squared error of the fit was less than 10% and the squared correlation coefficient between the DPOAE signal and the fitted function was greater than 0.9. A detailed description of the PBF algorithm can be found elsewhere (Zelle *et al.*, 2013; Zelle *et al.*, 2015b) and six examples are given in the supplementary material.^1^

Denoting the amplitudes of the nonlinear-distortion and coherent-reflection components by ${\hat{P}}_{1}$ and ${\hat{P}}_{2}$, respectively, the I/O functions were grouped into fine-structure (FS) affected and no-FS affected, depending on whether their amplitude ratio, ${\hat{P}}_{2}/{\hat{P}}_{1}$, was greater than 0.25 at L_2_ = 45 dB SPL. This lower bound corresponds to a maximal amplitude error due to wave interference of 2.5 dB. Depending on the relative phase difference, $\Delta \varphi =\varphi_{2}-\varphi_{1}$, between the extracted components, FS-affected I/O functions were further classified into constructive interference (Δφ=0°±45°), destructive interference (Δφ=180°±45°), and quadrature otherwise. Despite its expected dependence on stimulus level (He and Schmiedt, 1993; Zelle *et al.*, 2015a), the interference type was evaluated at only one pair of primary-tone levels (L_2_ = 45 dB SPL) and, consequently, only one type was assigned to an I/O function.

## III. RESULTS

### A. DPOAE I/O-functions

The proportion of DPOAE I/O functions with three or more points satisfying the 10-dB SNR criterion (Sec. II D and II E), called here “computable” DPOAE I/O functions, was higher for continuous stimulation than for short-pulse stimulation; namely, 92.1% (302/328) as opposed to 83.5% (274/328) (Table I). Applying the acceptance criteria based on the parameters $r_{I/O}^{2}$, $s_{I/O}$, and $\sigma_{EDPT}$ (Sec. II E) derived from the linear regression analysis, the number of I/O functions accepted for auditory-threshold estimation, N_a_, decreased from 274 to 237 (86.5%) in the case of short-pulse DPOAEs and from 302 to 238 (78.8%) for continuous DPOAEs; that is, these acceptance criteria resulted in a greater proportion of the continuous DPOAEs being rejected. However, incorporating the HLC algorithm (Sec. II E) before performing linear regression resulted in the two stimulus conditions having similar acceptance rates − 92.7% (254/274) for short-pulse stimulation and 91.4% (276/302) for continuous stimulation.

**TABLE I.** Acceptance rates and evaluation parameters of I/O functions for both stimulus paradigms with and without high-level correction (HLC; Sec. II E). N: Number of I/O functions with at least three DPOAEs satisfying the 10-dB SNR criterion (Sec. II D). N_a_: Number of EDPTs complying with the regression-fit acceptance criteria that $r_{I/O}^{2}$ ≥ 0.8, $\sigma_{EDPT}$ ≤ 10 dB, $s_{I/O}$ ≥ 0.2 μPa/dB SPL, and  EDPT  ≥ −10 dB SPL (Sec. II E). Acceptance rates relative to N are given in parentheses. ${\tilde{r}}_{I/O}^{2}$, ${\tilde{s}}_{I/O}$, and ${\tilde{\sigma }}_{EDPT}$: Median values for the pooled evaluation parameters with interquartile range given in square brackets.

	Short-pulse DPOAE		Continuous DPOAE	
	No HLC	HLC	No HLC	HLC
N	274/328 (83.5%)		302/328 (92.1%)	
N_a_	237 (86.5%)	254 (92.7%)	238 (78.8%)	276 (91.4%)
${\tilde{r}}_{I/O}^{2}$	0.97 [0.04]	0.98 [0.03]	0.96 [0.06]	0.97 [0.04]
${\tilde{\sigma }}_{EDPT}$ (dB)	1.77 [1.86]	1.54 [1.67]	1.93 [2.13]	1.53 [1.61]
${\tilde{s}}_{I/O}\;\left(\frac{\mu Pa}{dB\;SPL}\right)$	2.61 [2.53]	2.76 [2.53]	2.45 [2.19]	2.53 [2.52]

Table I also shows the median values of the evaluation parameters for the accepted I/O functions, denoted as ${\tilde{r}}_{I/O}^{2}$, ${\tilde{\sigma }}_{EDPT}$, and ${\tilde{s}}_{I/O}$, for both acquisition paradigms with and without the HLC algorithm. A two-sided Wilcoxon rank sum test was applied to the pooled evaluation parameters for all frequencies and subjects to identify differences between stimulus paradigms and variations due to the HLC algorithm. The HLC algorithm yielded small but significant improvements in $r_{I/O}^{2}$ and $\sigma_{EDPT}$ for both acquisition paradigms, with *p* < 0.0001 for ${\tilde{r}}_{I/O}^{2}$ and *p* < 0.01 for ${\tilde{\sigma }}_{EDPT}$. The slope parameter, $s_{I/O}$, was not changed significantly by the HLC algorithm (continuous DPOAE: *p* = 0.85; short-pulse DPOAE: *p* = 0.51). None of the evaluation parameters exhibited significant differences between acquisition paradigms for the unmodified I/O functions (${\tilde{r}}_{I/O}^{2}$: *p* = 0.12; ${\tilde{\sigma }}_{EDPT}$: *p* = 0.74; ${\tilde{s}}_{I/O}$: *p* = 0.30), nor when corrected for high-level deviations from the expected straight-line behavior (${\tilde{r}}_{I/O}^{2}$: *p* = 0.14; ${\tilde{\sigma }}_{EDPT}$: *p* = 0.74; ${\tilde{s}}_{I/O}$: *p* = 0.13).

### B. Interference effects

**FIG. 4.**
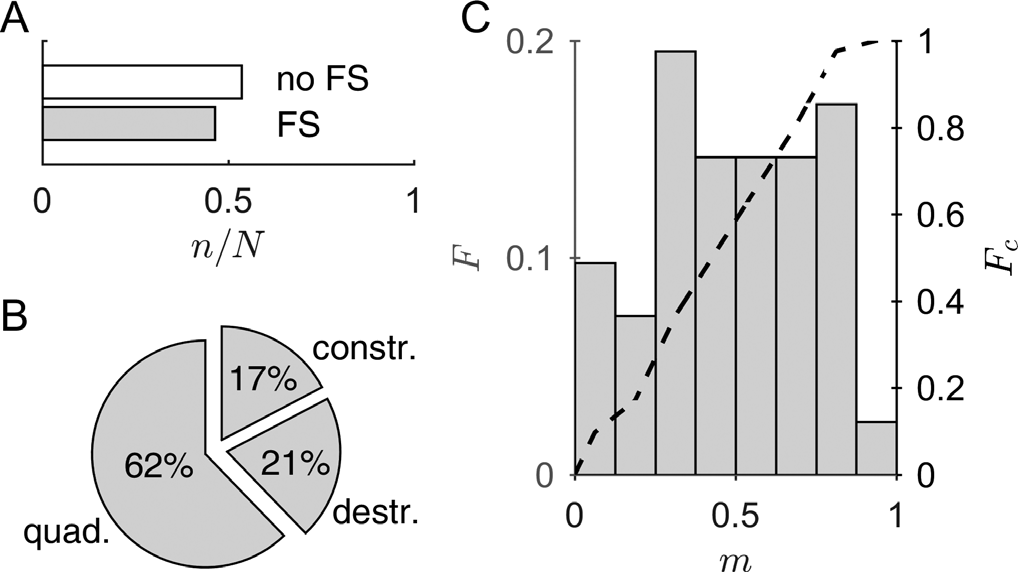
Statistics associated with fine structure. (A) Number of I/O functions, n, relative to the total number of computable I/O functions for the pooled data, N, divided into two groups according to the presence of underlying fine structure (FS) at L_2_ = 45 dB SPL. Definition of the FS group: Amplitude of the coherent-reflection component greater than or equal to 25% of the amplitude of the nonlinear-distortion component; conversely, for the no-FS group. (B) The FS-group was further divided into three types of interference: quadrature (*quad*.), destructive (*destr*.), and constructive (*constr*.), depending on the relative phase difference between the two DPOAE components (Sec. II F). (C) Normalized histogram, *F*, of the relative occurrence, *m*, of FS-affected I/O functions across subjects. *m* tends toward a uniform distribution (one-sample Kolmogorov-Smirnov test, *p* = 0.14). The dashed line corresponds to the empirical distribution function, *F*_c_. It indicates that 41.5% of the subjects have at least half of their computable I/O functions belonging to the fine-structure group.

According to PBF decomposition of the short-pulse DPOAE responses at a level L_2_ ≥ 45 dB SPL (Sec. II F), 46.4% (127/274) of the computable I/O functions exhibited a coherent-reflection component, $p_{2}(t)$, with an amplitude, ${\hat{P}}_{2}$, greater than or equal to 25% of the amplitude, ${\hat{P}}_{1}$, of the nonlinear-distortion component, $p_{1}(t)$ [Fig. 4(A)]. The associated I/O-functions were rated as FS-affected and further grouped into the three underlying interference types in compliance with the relative phase difference between the two DPOAE components. Referring to Fig. 4(B), 62.2% (79/127) of the I/O functions exhibited quadrature, i.e., a phase difference close to 90°, while destructive and constructive interference were less frequent with 20.5% (26/127) and 17.3% (21/127), respectively. These proportions slightly differ from the values expected for phase differences uniformly distributed across frequency; namely, 50% for quadrature and 25% each for destructive and constructive interference. Figure 4(C) depicts the normalized histogram, *F* (gray bars), of the distribution of the relative occurrence, *m*, of FS-affected I/O functions across subjects. For each subject, *m* was computed as the number of FS-affected I/O functions divided by the number of computable I/O functions. The histogram indicates that the proportion of I/O functions with an interfering coherent-reflection component tends to be uniformly distributed across subjects. The empirical distribution function, *F*_c_ [dashed line in Fig. 4(C)], yields a value of *F*_c_(*m*) = 0.585 for *m* = 0.5, implying that in 41.5% of the subjects more than half of the computable I/O functions are FS-affected. There was no correlation between the ratio ${\hat{P}}_{2}/{\hat{P}}_{1}$ and L_BT_ (r = 0.00; *p* = 0.996). The portion of FS-affected I/O functions was similar for normal-hearing (L_BT_ < 20 dB HL) and hearing-impaired thresholds with 46.4% (109/235) and 46.2% (18/39), respectively, suggesting that an interfering coherent-reflection component may also occur in hearing-impaired subjects.

The impact of a pronounced coherent-reflection component on the growth behavior and shape of the I/O functions was quantified with $r_{I/O}^{2}$, using all computable DPOAE I/O functions without applying the HLC algorithm. In the case of FS-affected I/O functions, the median value for the short-pulse DPOAEs, ${\tilde{r}}_{I/O}^{2}$ = 0.96, was significantly larger than that for the continuous DPOAEs, ${\tilde{r}}_{I/O}^{2}$ = 0.94 (one-sided Wilcoxon rank sum test, *p* = 0.03), with corresponding interquartile ranges (IQR) of 0.06 and 0.12. For the continuous DPOAEs, 38.6% of the I/O functions exhibited $r_{I/O}^{2}$ < 0.9, whereas it was only 18.9% for short-pulse DPOAEs. For the non-FS-affected I/O functions, ${\tilde{r}}_{I/O}^{2}$ for the short-pulse DPOAEs (${\tilde{r}}_{I/O}^{2}$ = 0.96, IQR = 0.05) was not significantly different to that for the continuous DPOAEs (${\tilde{r}}_{I/O}^{2}$ = 0.96, IQR = 0.08; two-sided Wilcoxon rank sum test, *p* = 0.396) and the proportion of I/O functions with $r_{I/O}^{2}$ < 0.9 was similar (short-pulse DPOAEs: 19.9%; continuous DPOAEs: 24.0%).

**FIG. 5.**
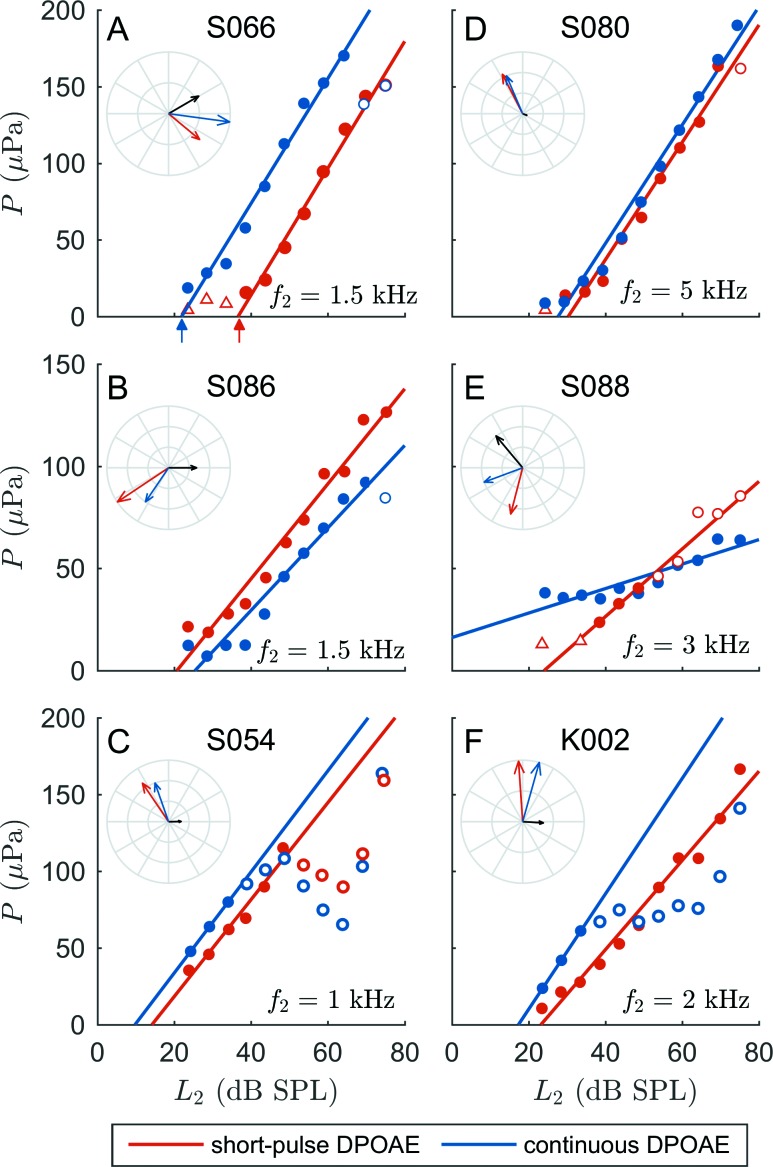
DPOAE I/O functions based on continuous (blue dots) and short-pulse (red dots) stimulation for six subjects. The intersections of the linear regression lines (blue and red lines) with the abscissa define the EDPTs (exemplarily indicated by the blue and red arrows in A). Empty triangles correspond to DPOAEs not complying with the 10-dB SNR criterion (Sec. II D), while empty circles depict data points excluded from the regression analysis by high-level correction (HLC; Sec. II E). Insets show diagrams of the phasors $P_{1}$ and $P_{2}$ (both rotating at 2πf_DP_ rad/s) of the nonlinear-distortion (red arrows) and coherent-reflection (black arrows) components extracted with PBF decomposition at L_2_ = 45 dB SPL (Zelle *et al.*, 2013; Zelle *et al.*, 2015b), as well as the phasor sum $P_{c}=P_{1}+P_{2}$ (blue arrow), which provides an estimate of the continuous DPOAE phasor and serves as a comparison with the measured continuous DPOAE. The short-pulse DPOAE signals together with the statistical parameters associated with the PBF decomposition are given in the supplementary material.^1^ The phasor amplitudes of $P_{1}$ and $P_{2}$ in A, B, E, and F indicate pronounced coherent-reflection components capable of altering I/O functions depending on the phase difference between $P_{1}$ and $P_{2}$. The coherent-reflection component in A enhances the amplitude of $P_{c}$, shifting the I/O function toward lower L_2_ levels. In contrast, in B, destructive interference shifts the continuous I/O function toward higher L_2_ values. In E and F, interference conditions vary considerably with stimulus level yielding an unreasonably flat I/O function in E and considerable deformations in F. Data in C and D do not contain references to pronounced coherent-reflection components. However, C depicts deformations in both I/O functions; these data points (empty circles) were detected by the HLC algorithm as being systematic deviations from the straight-line growth evident at low intensities and were, therefore, excluded from the regression analysis.

Figure 5 illustrates I/O functions for various types of interference patterns recorded for continuous (blue dots) and short-pulse (red dots) stimulation for six subjects. EDPTs used for auditory-threshold estimation, defined as the intersection of the linear regression lines (blue and red lines) with the abscissa, are exemplarily indicated in Fig. 5(A) by blue and red arrows. Circles represent DPOAE amplitudes excluded from the computation of the linear regression lines by the HLC algorithm (Sec. II E). Insets show phasor diagrams illustrating the phasors $P_{1}={\hat{P}}_{1}e^{i\varphi_{1}}$ (red arrow) and $P_{2}={\hat{P}}_{2}e^{i\varphi_{2}}$ (black arrow) associated with the nonlinear-distortion and coherent-reflection components, respectively. Amplitudes $\hat{P}$ and phases φ correspond to the parameters extracted from the short-pulse DPOAE responses at L_2_ = 45 dB SPL using PBF decomposition (Sec. II F; supplementary material).^1^ The blue arrow is the phasor sum of the two extracted components, $P_{c}=P_{1}+P_{2}$, and represents an estimate of the phasor for DPOAEs measured with continuous stimulation.

The impact of wave interference on the shape of the I/O functions varies according to the underlying type of interference defined by the phase difference, $\Delta \varphi =\varphi_{2}-\varphi_{1}$, and the relative phasor amplitudes, ${\hat{P}}_{2}/{\hat{P}}_{1}$. Figures 5(A) and 5(C) show two examples of phase difference close to quadrature. In Fig. 5(A), the contribution of the coherent-reflection component is relatively large with ${\hat{P}}_{2}/{\hat{P}}_{1}$ = 0.86, and leads to an increase of the amplitude, ${\hat{P}}_{c}$, of the phasor sum due to the phase difference of Δφ= −290°; these relative values explain the shift of the I/O function for continuous DPOAEs toward lower L_2_ values. In contrast, the example in Fig. 5(C) illustrates the case of a relatively small coherent-reflection component (${\hat{P}}_{2}/{\hat{P}}_{1}$ = 0.27) with quadrature phase tending to destructive interference (Δφ = −123°), which yielded a (small) decrease in the amplitude, ${\hat{P}}_{c}$. In other words, the larger amplitudes observed experimentally in this example for the continuous DPOAEs at low intensities cannot be due to such wave interference; presumably other factors are influencing the response, such as noise or calibration differences between the two measurements. Figure 5(B) shows an example of destructive interference (${\hat{P}}_{2}/{\hat{P}}_{1}$ = 0.46; Δφ = 146°) shifting the continuous I/O function toward higher L_2_ values, while Fig. 5(D) lacks a significant coherent-reflection component (${\hat{P}}_{2}/{\hat{P}}_{1}$ = 0.10) and both I/O functions nearly superimpose. Figure 5(E) is an example of DPOAE components with similar amplitude (${\hat{P}}_{2}/{\hat{P}}_{1}$ = 0.87), showing a pronounced variation of the interference condition with increasing stimulus level. While quadrature dominates at L_2_ = 45 dB SPL (Δφ = −126°), constructive interference prevails at low primary-tone levels and destructive interference begins at L_2_ ≥ 60 dB SPL. The I/O functions shown in Figs. 5(C) and 5(F) exhibit distinct deviations from the expected linear relationship. The HLC algorithm reduces the impact of these deformations by excluding DPOAE amplitudes for L_2_ values exceeding a threshold level determined by the algorithm. In Fig. 5(C), both short-pulse and continuous DPOAE I/O functions show a notch around 60 dB SPL, whereas only the continuous data differs from the linear relationship in Fig. 5(F). All three “deformed” I/O functions in Figs. 5(C), 5(E), and 5(F) would yield considerably lower EDPT values, if these data points were to be included in the linear regression fit.

### C. Relation between behavioral thresholds and EDPTs

**FIG. 6.**
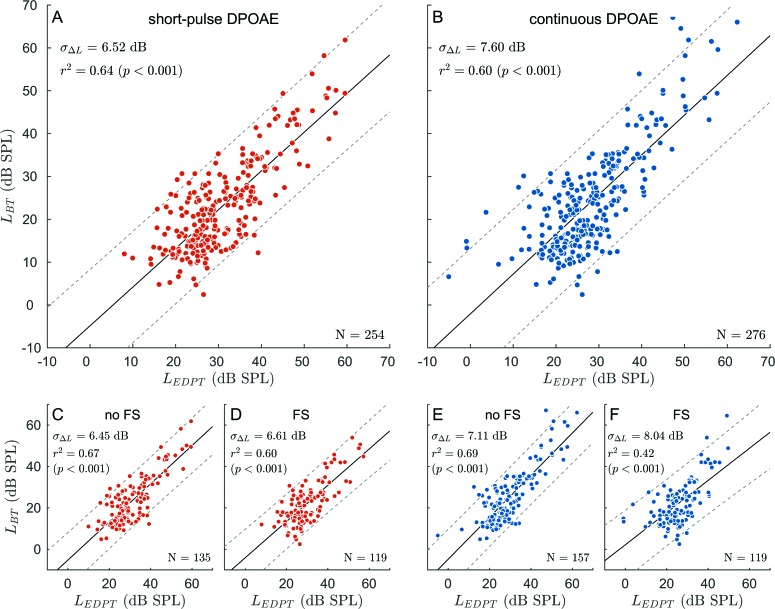
Correlation of the behavioral thresholds, L_BT_, with estimated distortion-product thresholds, L_EDPT_, for short-pulse (A) and continuous (B) stimulation pooled over all subjects and frequencies. (C) and (D) Scatter plots, for short-pulse DPOAEs, for the two subsets associated with I/O functions with (D) and without (C) fine-structure (FS) at 45 dB SPL. (E) and (F) Equivalent representation for continuous DPOAEs. Black solid and gray dashed lines in all panels represent the corresponding regression lines and the 95% confidence intervals, respectively. Regression parameters are given in Table II. In general, EDPTs derived with short-pulse DPOAE I/O functions relate more accurately to BTs with less scatter compared to EDPTs based on continuous DPOAEs [(A) and (B)]. A major reason for the performance differences between the stimulus paradigms is interference between the two DPOAE components, which becomes evident when comparing the scatter plots of the FS-affected groups [(D) and (F)] particularly for continuous DPOAEs.

For both acquisition paradigms, EDPTs were related to behavioral thresholds (BTs) estimated by the adapted version of Békésy tracking audiometry (Sec. II C). Figure 6 shows the level of the Békésy threshold, L_BT_, as a function of the EDPT level, L_EDPT_, for the high-level corrected data comprising all subjects and all frequencies for short-pulse [Fig. 6(A)] and continuous [Fig. 6(B)] stimuli. For both stimulus paradigms, the BTs show a significant correlation with EDPTs, with the short-pulse data presenting slightly higher squared correlation coefficients ($r^{2}$ = 0.64; *p* < 0.001) than EDPTs based on continuous DPOAEs ($r^{2}$ = 0.60; *p* < 0.001). Regression analysis between L_BT_ and L_EDPT_ reveals a linear relationship, enabling estimated hearing thresholds (EHT), L_EHT_, to be derived from EDPTs according to

$$L_{EHT}=aL_{EDPT}+b.$$ (7)

The fit parameters a and b are given in Table II for both stimulus paradigms, averaged for each stimulus frequency. All I/O functions were subjected to the HLC algorithm (Sec. II E) before the regression analysis.

**TABLE II.** Regression parameters for estimating hearing thresholds from EDPTs [Eq. (7)] for both stimulus paradigms. DPOAE I/O functions were subjected to high-level correction (HLC; Sec. II E). *f*_2_: Stimulus frequency and, in the case of DPOAEs, the frequency of the second primary tone. Parameters are pooled across frequency, denoted by the row label “1,…,8,” and also partitioned and pooled across frequency according to the absence or presence of fine structure (FS) at L_2_ = 45 dB SPL, denoted by the row labels “no FS” and “FS,” respectively. N_a_: Number of accepted EDPTs (Sec. II E, and Table I). $r^{2}$: Squared correlation coefficient [Eq. (7)]. $\sigma_{\Delta L}$: Standard deviation of the differences between $L_{EHT}$ [Eq. (7)] and $L_{BT}$. a and b: Slope and constant parameters of the linear regression line [Eq. (7)]. All correlations were significant (*p* < 0.001), except for short-pulse EDPTs at *f*_2_ = 1 kHz (*p* = 0.07) and 8 kHz (*p* = 0.22). For both stimulus paradigms, at *f*_2_ = 1 and 8 kHz, the slope values are those from the pooled data (row label “1,…,8”) because of limited dynamic range in the DPOAE I/O function at these two frequencies.

	Short-pulse DPOAE					Continuous DPOAE				
$f_{2}$ (kHz)	N_a_	$r^{2}$	$\sigma_{\Delta L}$ (dB)	*a*	*b* (dB SPL)	N_a_	$r^{2}$	$\sigma_{\Delta L}$ (dB)	*a*	*b* (dB SPL)
1	27	0.13	6.94	(0.90 ± 0.04)	−7.67 ± 1.35	35	0.33	7.07	(0.93 ± 0.05)	−7.36 ± 1.21
1.5	34	0.58	5.58	0.66 ± 0.10	−0.49 ± 2.88	35	0.57	7.44	0.80 ± 0.12	−1.15 ± 3.30
2	39	0.74	5.82	1.00 ± 0.10	−8.63 ± 3.23	38	0.76	5.60	1.00 ± 0.10	−5.36 ± 2.79
3	35	0.79	4.93	0.96 ± 0.09	−7.51 ± 2.71	32	0.60	7.01	0.80 ± 0.12	0.76 ± 3.38
4	36	0.67	6.83	1.02 ± 0.12	−8.54 ± 4.17	39	0.77	6.92	1.09 ± 0.10	−7.00 ± 3.29
5	32	0.63	6.37	0.97 ± 0.14	−4.05 ± 4.84	34	0.58	6.96	1.04 ± 0.16	−1.83 ± 5.02
6	32	0.60	5.93	0.82 ± 0.13	0.04 ± 4.61	32	0.66	6.09	1.32 ± 0.17	−11.35 ± 5.51
8	19	0.09	7.95	(0.90 ± 0.04)	−2.92 ± 1.87	31	0.36	8.82	(0.93 ± 0.05)	0.75 ± 1.60
1,…,8	254	0.64	6.52	0.90 ± 0.04	−4.9 ± 1.37	276	0.60	7.60	0.93 ± 0.05	−2.11 ± 1.37
no FS	135	0.67	6.45	0.92 ± 0.06	−5.32 ± 1.83	157	0.69	7.11	1.02 ± 0.05	−4.91 ± 1.70
FS	119	0.60	6.61	0.88 ± 0.07	−4.26 ± 2.08	119	0.42	8.04	0.78 ± 0.08	2.09 ± 2.30

**FIG. 7.**
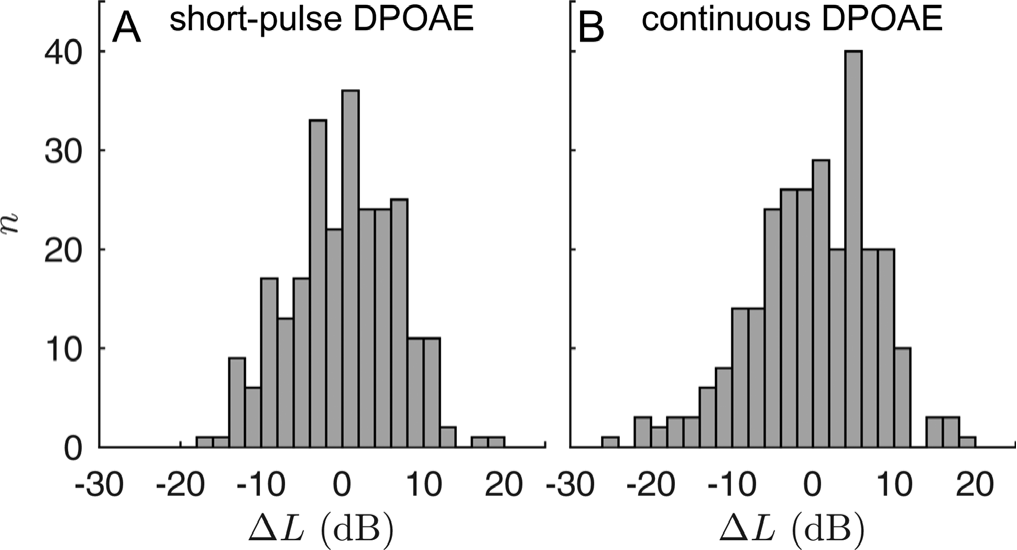
Histograms of the difference ΔL between L_EHT_ given by Eq. (7) and L_BT_, for short-pulse (A) and continuous (B) acquisition. The data are normally distributed with zero mean and standard deviations of 6.52 dB (one-sample Kolmogorov-Smirnov test, *p* = 0.82) and 7.60 dB (*p* = 0.38) for short-pulse and continuous stimulation, respectively. The variance of the differences for the short-pulse data is significantly lower than that of the continuous data (one-sided F-test, *p* < 0.01).

The accuracy of the auditory-threshold estimation procedure was assessed using the standard deviation, $\sigma_{\Delta L}$, of the differences between $L_{EHT}$ and $L_{BT}$, both for each stimulus frequency and also for all frequencies of the pooled data (Table II). Figure 7 shows the histograms of ΔL for short-pulse [Fig. 7(A)] and continuous [Fig. 7(B)] stimulation. Pooled over all frequencies and subjects, the standard deviation, $\sigma_{\Delta L}$ = 6.52 dB, for the short-pulse data was significantly less than the $\sigma_{\Delta L}$ = 7.60 dB for the continuous data (one-sided F-test for variances, *p* < 0.01). To estimate the impact of two-component interference on the accuracy of $L_{EHT}$, the data were partitioned into FS- and non-FS-affected I/O functions. Figures 6(C)–6(F) show the scatter plots of $L_{BT}$ as a function of $L_{EDPT}$ for the two groups and both DPOAE paradigms. Comparing the no-FS groups between the two DPOAE paradigms reveals that there is no statistically significant difference in the variance of ΔL between the short-pulse and the continuous data [two-sided F-test, *p* = 0.24; Figs. 6(C) and 6(E)]. In contrast, EDPTs from the FS group exhibit a significantly smaller variance if recorded with short-pulse stimuli compared to continuous stimuli (one-sided F-test, *p* = 0.02), which is also evidenced by the lower standard deviation, $\sigma_{\Delta L}$ = 6.61 dB, for short-pulse DPOAEs [Fig. 6(D)] compared to $\sigma_{\Delta L}$ = 8.04 dB for continuous DPOAEs [Fig. 6(F)].

The smaller number of accepted EDPTs for auditory-threshold estimation in the case of short-pulse stimulation (Table I, row labelled N_a_ and columns labelled HLC) results mainly from the lower acceptance rates at *f*_2_ = 1 and 8 kHz of only 65.9% and 46.4%, respectively (Table II). While continuous DPOAEs enhanced the acceptance rate for these frequencies, they did not yield a more accurate threshold estimate, particularly at *f*_2_ = 8 kHz where $\sigma_{\Delta L}$ = 8.82 dB. However, EDPTs based on continuous DPOAEs can be more precisely related to subjective thresholds at *f*_2_ = 2 kHz ($\sigma_{\Delta L}$ = 5.60 dB). Short-pulse EDPTs offered more accurate auditory-threshold estimates for *f*_2_ from 1.5 to 3 kHz and at 6 kHz, where all standard deviations were below 6 dB. The best performance was achieved with short-pulse EDPTs at *f*_2_ = 3 kHz with $\sigma_{\Delta L}$ = 4.93 dB.

**FIG. 8.**
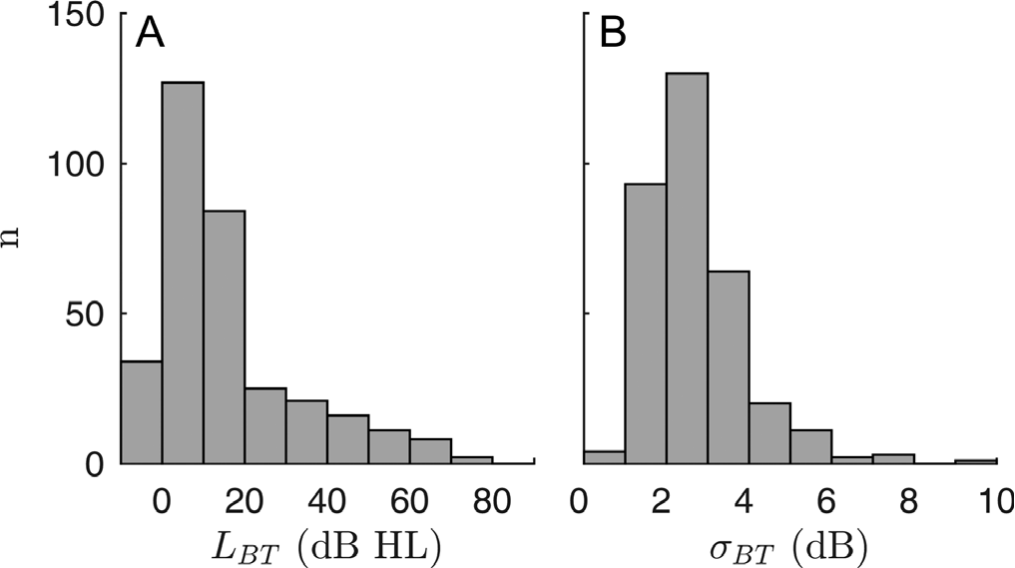
Statistics of behavioral thresholds (BT) estimated by a modified version of Békésy tracking audiometry (Sec. II C). (A) Histogram of BT levels (L_BT_) in dB HL. 74.7% of the thresholds were below 20 dB HL. Only 3.1% of the pooled data presented BTs higher than 60 dB HL and, therefore, presumably almost complete loss of cochlear amplification. (B) Histogram of the standard deviations, $\sigma_{BT}$, of L_BT_. The median value of $\sigma_{BT}$ was ${\tilde{\sigma }}_{BT}$ = 2.37 dB, suggesting accurate and reproducible estimates of L_BT_, as required when L_BT_ is used as a reference for establishing the reliability of EDPTs as an objective estimator of auditory thresholds.

74.7% (245/328) of all thresholds measured with Békésy audiometry were below 20 dB HL [Fig. 8(A)]. I/O functions recorded at frequencies with normal hearing showed high acceptance rates, with 95.9% (235/245) and 89.4% (219/245) for continuous and short-pulse stimuli, respectively. The number of I/O functions accepted for threshold estimation remains large for moderately elevated BTs in the range of 20 < L_BT_ ≤ 40 dB HL with 73.9% (34/46) and 71.7% (33/46), respectively, but declines notably at thresholds above 40 dB HL to 18.9% (7/37) for the continuous data and 5.4% (2/37) for the short-pulse data. Figure 8(B) depicts the histogram of the standard deviations of the BTs computed from the three consecutive recordings for each subject. The median value of the standard deviations was ${\tilde{\sigma }}_{BT}$ = 2.37 dB (IQR = 1.25 dB). This relatively small range means that the subjective thresholds used as the basis for determining the accuracy of the objectively derived auditory thresholds is accurate and reproducible.

### D. Individual threshold estimation

**FIG. 9.**
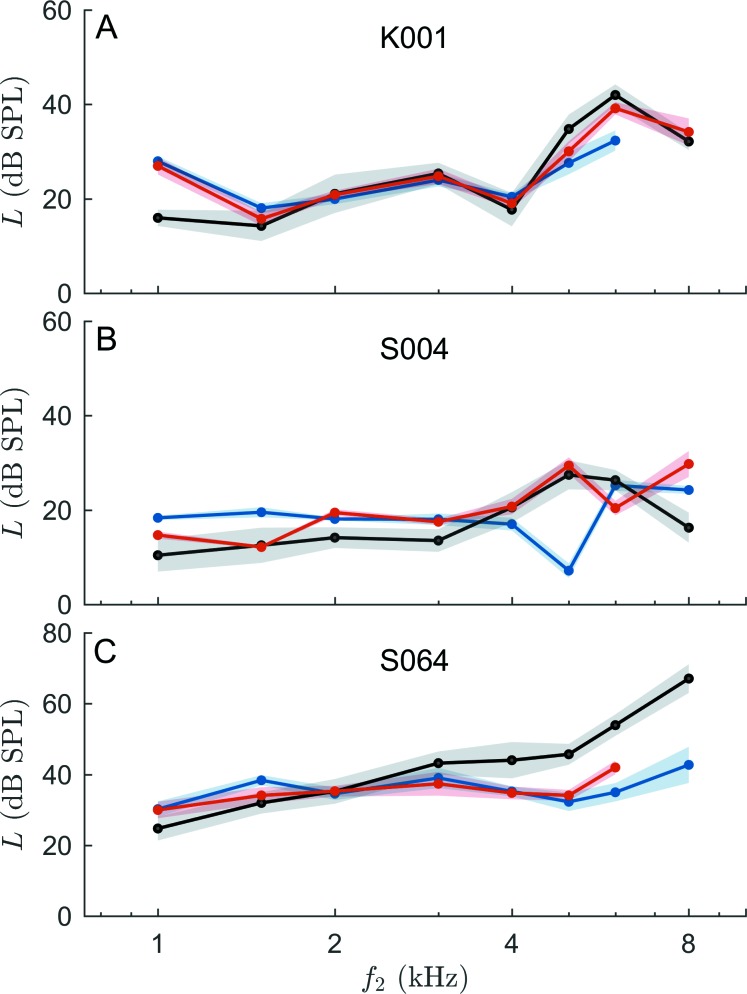
Individual auditory-threshold estimation for three subjects utilizing estimated hearing thresholds (EHTs) computed from EDPTs according to Eq. (7) for short-pulse (red; *pulsed*) and continuous (blue; *cont.*) DPOAEs. Black lines: Behavioral thresholds (BTs). Shaded areas: Standard deviations of EDPTs, $\sigma_{EDPT}$, derived from the linear regression analysis of the DPOAE I/O function, and of BTs, $\sigma_{BT}$, derived from the three consecutive Békésy measurements. In general, EHTs match BTs closely, but the continuous data are prone to large differences between EHTs and BTs (e.g., at 5 kHz in B) due to wave interference. Both stimulus paradigms exhibit large deviations from BTs for *f*_2_ = 1 kHz (A) or 8 kHz (B and C), which is also reflected in the statistics given in Table II. Evaluation parameters (see Sec. III D for definitions) from subjects K001 (A), S004 (B), and S064 (C) are as follows. For short-pulse EHTs: (A) $\sigma_{\Delta L,ind}$ = 4.67 dB, $\Delta L_{max}$ = 11.00 dB; (B) $\sigma_{\Delta L,ind}$= 5.58 dB, $\Delta L_{max}$ = 13.50 dB; (C) $\sigma_{\Delta L,ind}$ = 6.95 dB, $\Delta L_{max}$ = 11.95 dB. For continuous EHTs: (A) $\sigma_{\Delta L,ind}$ = 7.36 dB, $\Delta L_{max}$ = 11.94 dB; (B) $\sigma_{\Delta L,ind}$= 9.26 dB, $\Delta L_{max}$ = 19.12 dB; (C) $\sigma_{\Delta L,ind}$ = 11.26 dB, $\Delta L_{max}$ = 25.32 dB.

Exploiting the linear relationship between BTs and EDPTs enables estimation of hearing thresholds using Eq. (7), which provides an indication of the integrity of the biomechanical part of the hearing system. Plotting the estimated hearing threshold (EHT) as function of *f*_2_ yields an objectively measured audiogram for each subject. Figure 9 shows examples for objective audiograms based on continuous (blue line) and short-pulse (red line) DPOAEs for three subjects. For comparison, BTs are shown in black. Shaded areas correspond to $\sigma_{EDPT}$ and $\sigma_{BT}$, respectively. The accuracy of the individual auditory-threshold estimates was quantified with the standard deviation $\sigma_{\Delta L,ind}$ of the differences between $L_{EHT}$ and $L_{BT}$ across all frequencies for that subject. In general, both stimulus paradigms yielded objective audiograms matching the subjective threshold closely, with the short-pulse paradigm producing significantly smaller mean individual estimation errors, ${\bar{\sigma }}_{\Delta L,ind}$ = 5.44 ± 2.16 dB, than the continuous paradigm, 6.38 ± 2.57 dB (one-sided t-test, *p* = 0.006). Despite using the HLC algorithm, continuous DPOAEs remained prone to large deviations due to two-component interference—they result in maximum deviations, $\Delta L_{max}$, between subjective and estimated thresholds of up to 25.0 dB [cf. Fig. 9(B)], whereas I/O functions based on short-pulse DPOAEs yielded maximum errors not larger than 18.4 dB. On average, short-pulse EDPTs yielded ${\bar{\Delta L}}_{max}$ in the objective audiograms of 10.39 ± 3.34 dB, which is slightly but significantly less than 12.20 ± 5.13 dB obtained using the continuous EDPTs (one-sided t-test, *p* = 0.003).

## IV. DISCUSSION

DPOAE I/O functions based on the extracted nonlinear-distortion components enable the estimation of auditory thresholds with high accuracy and, therefore, offer a promising approach for objectively assessing hearing status. Section IV A assesses the efficacy of short-pulse stimuli for separating the two DPOAE components and is followed by a discussion (Sec. IV B) of error sources for the regression analysis resulting from systematic deviation from a straight-line semi-logarithmic DPOAE I/O function. The next two sections compare the acceptance rate of the DPOAE I/O functions for the purpose of EDPT estimation (Sec. IV C) and the accuracy of the auditory-threshold estimate (Sec. IV D) with previously published results. Section IV E discusses the accuracy of EDPTs for assessing hearing status. The concluding section (Sec. IV F) discusses implications of the current findings for employing DPOAE I/O functions as a clinical tool.

### A. Separation of DPOAE components

Short-pulse stimulation enabled the separation of the two DPOAE components by means of onset decomposition (OD). The fidelity of the separation can be directly assessed in DPOAE responses with destructive interference, where both components become readily distinguishable in the time signal [Fig. 3(A)] and in the instantaneous phase (supplementary material).^1^ However, for other interference conditions, such as quadrature or constructive interference, the DPOAE components are not always easily distinguishable. In such cases, comparison with other methods allows assessment of the quality of the algorithms presented here.

Vetešník *et al.* (2009) acquired high-resolution DP-grams to compare OD with the time-windowing technique by Kalluri and Shera (2001) and showed that OD successfully reduced DPOAE fine structure in a frequency range of *f*_2_ = 1.5,…, 2.5 kHz. That study employed a pre-defined sampling instant between 8 to 10 ms relative to the *f*_2_ onset. However, the optimal sampling instant for OD was found to decrease with increasing stimulus level. This finding is in accordance with other studies showing that latencies of DPOAEs vary considerably with stimulus frequency and level (Stover *et al.*, 1996; Zelle *et al.*, 2015b). Recently, the OD technique was extended to frequencies of *f*_2_ = 1,…, 8 kHz, to extract the nonlinear-distortion component from short-pulse DPOAEs using pre-defined, frequency-specific sampling instants (Zelle *et al.*, 2015a). That algorithm yielded a considerably smoother dependence of DPOAE amplitude on stimulus levels L_1_ and L_2_ compared to data from continuous stimulation. This result indicated successful extraction of the amplitude of the nonlinear-distortion component by OD. Alternatively, the time course of the underlying DPOAE components can be visualized with pulse basis functions (PBFs) in the time domain by fitting the DPOAE short-pulse response to a mathematical model that mimics the superposition of the components (Zelle *et al.*, 2013). This technique has the advantage that both the amplitudes and the phases of each component can be extracted.

The modified OD approach used in the present study, in which the onset of the DPOAE signal was detected objectively by an automated algorithm (Sec. II D), was additionally compared to extraction by PBF decomposition for short-pulse stimuli with *f*_2_ = 1,…, 4 kHz in six subjects (data not shown). Both methods provide a generally reliable extraction of the nonlinear-distortion component, as supported by the almost complete removal of fine structure. OD slightly underestimated the amplitude of the nonlinear-distortion component because it samples the DPOAE signal prior to its maximum. PBF decomposition resulted in extracted components, which reproduced known properties of the two DPOAE components reported by others (Shera and Guinan, 1999). However, successful decomposition into PBFs requires the absence of additional signals in the recordings which might otherwise hinder separation, e.g., SOAEs or further DPOAE components (Zelle *et al.*, 2015a; their Fig. 5 and Fig. 6). In contrast, component extraction using OD does not depend on extensive assumptions to model the DPOAE signal and, currently, proves to be the more robust technique.

### B. Irregularities in DPOAE I/O functions and deviation from linearity

The squared correlation coefficient $r_{I/O}^{2}$ between DPOAE amplitudes and L_2_ values was used to test I/O functions for the expected straight-line semi-logarithmic relationship. One major cause for deviation from linearity is interference between the DPOAE components (Mauermann and Kollmeier, 2004; Dalhoff *et al.*, 2013) which, in the case of the fine-structure (FS) group, is indicated by the significantly higher $r_{I/O}^{2}$ when using short-pulse as opposed to continuous stimulation [Figs. 6(D) and 6(F), respectively]. For the no-FS group, there were no significant differences in $r_{I/O}^{2}$ between stimulus paradigms [Figs. 6(C) and 6(E)], whereas for the FS group, the continuous DPOAE data yielded a higher interquartile range of $r_{I/O}^{2}$ and a larger number of I/O-functions with $r_{I/O}^{2}$ < 0.9 as compared to the short-pulse DPOAE data. This observation adds further support to the notion that an interfering coherent-reflection component leads to deformations in a sizable number of I/O functions when using continuous DPOAEs. However, quantification of the interference with the aid of $r_{I/O}^{2}$ might underestimate the impact of the coherent-reflection component if its phase remains constant with varying L_2_. For example, Figs. 5(A) and 5(B) exhibit a distinct coherent-reflection component shifting the I/O functions along the abscissa without significantly altering its linear growth behavior. A variation of the interference condition with L_2_, as observed in shifts of minima and maxima in the DPOAE fine structure by others (He and Schmiedt, 1993; Kummer *et al.*, 1998), enlarges the deviation from linearity [Figs. 5(E) and 5(F)].

Nevertheless, even using short-pulse DPOAEs, approximately a fifth of I/O functions in the no-FS group exhibited $r_{I/O}^{2}$ < 0.9, indicating other potential sources for deviation from straight-line semi-logarithmic behavior. This observation was most pronounced for *f*_2_ ≤ 1.5 kHz. At these frequencies, short-pulse DPOAE recordings acquired at high stimulus levels revealed additional short-latency contributions, which became evident as considerably varying instantaneous phases and interference effects during DPOAE onset. These disturbances were similar to waveform complexities described by Martin *et al.* (2013), putatively indicating distributed DPOAE components generated basally to the *f*_2_-tonotopic place. For the cubic distortion product at *f*_DP_ = 2*f*_1_–*f*_2_ and frequency ratios *f*_2_/*f*_1_ = 1.2, the basally distributed contributions to the DPOAE signal were shown to exhibit horizontal phase banding implying a wave-fixed source (Martin *et al.*, 2010) and, hence, indicating a similar generation mechanism as for the nonlinear-distortion component. Some I/O functions presented saturating or decreasing DPOAE amplitudes at high stimulus levels, putatively reflecting compressional behavior of the cochlear amplifier or two-tone suppression between the primary tones in the case of L_1_ exceeding the optimal level for DPOAE generation (Robles and Ruggero, 2001). Optimized frequency-dependent stimulus levels have been shown to yield DPOAE I/O functions with linear growth over a wider intensity range compared to those based on the (frequency-independent) scissor paradigm, as well as larger slopes and less variation across stimulus frequency (Johnson *et al.*, 2006a; Zelle *et al.*, 2015a). However, for an individual subject, deviation from the optimal stimulus-level path defining L_1_ values as function of L_2_ to evoke maximum DPOAE amplitudes may yield deformations in the linear shape of semi-logarithmic I/O functions [e.g., Fig. 5(C)]. Furthermore, mathematical analysis (Lukashkin and Russell, 1999) has shown that deformations/irregularities may also be inherent to the nonlinear characteristics of the mechanosensitive channels in the OHC stereocilia which, dependent on the transducer operating point, can produce a notch in the DPOAE I/O function, as found for example in Fig. 5(C).

Several methods have been proposed to compensate for deviations of DPOAE amplitude from the expected straight-line semi-logarithmic relationship with L_2_: (1) fitting the data with different slopes depending on the DPOAE growth behavior (Goldman *et al.*, 2006; Neely *et al.*, 2009), (2) using a regression line weighted according to SNR and stimulus level (Oswald and Janssen, 2003), or (3) excluding those DPOAE data points with saturation behavior at high stimulus levels (Dalhoff *et al.*, 2013). The present study also employed saturation correction, but extended the algorithm of Dalhoff *et al.* (2013) by not only using the squared correlation coefficient to establish the quality of the linearization process but also the regression-line slope and the standard deviation of the EDPT. Using all three parameters to maximize quality avoids a preference for I/O functions with only a few DPOAE data points at low stimulus levels. The algorithm, called the high-level correction (HLC) algorithm (Sec. II E), is also effective at moderate stimulus levels, where it can reduce the impact of other sources of deformations and irregularities in the I/O functions, such as notches. The HLC algorithm yielded I/O functions with linear growth over a wider intensity range while minimizing the number of neglected data points [Figs. 5(C) and 5(F)].

### C. Acceptance rate of DPOAE I/O functions for threshold estimation

The number of DPOAE I/O functions complying with the objective evaluation criteria (Sec. II E), defined originally by Boege and Janssen (2002), relative to the number of computable I/O functions was similar for the two acquisition paradigms; the acceptance rates were 92.7% and 91.4% for short-pulse and continuous stimulation, respectively. These values were considerably higher than the acceptance rates of 68.5% reported by Boege and Janssen (2002) and 67.1% by Gorga *et al.* (2003) for a similar study design. One explanation for the lower acceptance rates in their studies may be the larger proportion of I/O functions at *f*_2_ ≤ 1 kHz and the higher number of hearing-impaired subjects than in the present study. Furthermore, for both acquisition paradigms, the HLC algorithm used in the present study appears to be another beneficial factor for the acceptance rate. While the acceptance rate for the corrected continuous DPOAE data was larger than the 84.7% reported by Dalhoff *et al.* (2013), there is a notable discrepancy between the short-pulse DPOAE data presented here and their pulsed data, namely, none of their I/O functions had to be excluded from the regression analysis after component separation and saturation correction. For comparison with the results of Dalhoff *et al.* (2013), the acceptance rate was re-evaluated for a subset of the present data by including only I/O functions at frequencies 1.5 ≤ *f*_2_ ≤ 3 kHz and only from the normal-hearing population (i.e., L_BT_ < 20 dB HL). For this subset, the acceptance rate for I/O functions recorded with short-pulse stimulation increases to 97.8% (90/92), which is close to the results of Dalhoff *et al.* (2013). Since SNR represents the major limiting factor for short-pulse data, the acceptance rate cannot be improved extensively by the HLC algorithm, in contrast to the continuous data.

### D. Relation between EDPTs and behavioral thresholds

Both DPOAE acquisition paradigms yielded EDPTs, which allowed the prediction of behavioral thresholds in a clinically relevant frequency range from *f*_2_ = 1 to 8 kHz with hitherto unreported accuracy of $\sigma_{\Delta L}$ = 6.52 dB and $\sigma_{\Delta L}$ = 7.60 dB, respectively, for short-pulse and continuous stimulation (Sec. III C; Table II). These values are notably smaller than those reported in previous studies utilizing continuous primary tones and stimulus levels based on the scissor paradigm. Boege and Janssen (2002) reported a value of 10.9 dB for a study population including normal-hearing and hearing-impaired ears, which was reproduced by Gorga *et al.* (2003) with 10.1 dB and Oswald and Janssen (2003) with 11.2 dB. Several reasons might be responsible for this poorer accuracy compared to the data presented here. First, contrary to the present work, in previous studies the DPOAE amplitude was estimated in the frequency domain from continuous recordings, which yielded amplitudes representing a superposition of the nonlinear-distortion and coherent-reflection components. When relating EDPTs from I/O functions to BTs associated with *f*_2_, the coherent-reflection component may induce errors in the threshold estimate. Therefore, extracting the nonlinear-distortion component from short-pulse DPOAE recordings, as was done in this study using OD, can be one reason for the increased accuracy. This suggestion is supported by the significantly smaller value of $\sigma_{\Delta L}$ = 6.52 dB for short-pulse data compared to the continuous data, both in the overall dataset ($\sigma_{\Delta L}$ = 7.60 dB) and in the fine-structure subset ($\sigma_{\Delta L}$ = 8.04 dB) (Table II). Furthermore, only I/O functions derived from continuous stimulation yielded unreasonably low EDPTs with L_EDPT_ < −10 dB SPL; such I/O functions were excluded from further analysis. Nonetheless, when using the continuous data, the present study also achieved a smaller estimation error than in those earlier studies. The difference might be related to the investigated population, which in the present study included fewer subjects with profound hearing loss than in previous studies. Since DPOAEs cannot assess the functional state of the auditory system beyond the cochlear amplifier, such as the inner hair cells (IHCs) or the auditory nerve, the observed EDPTs might underestimate BTs in cases of severe hearing loss. However, in the present study, only 11.3% of BTs exceeded 40 dB HL. Furthermore, a large number of hearing-impaired subjects would primarily yield an increased number of incomputable I/O functions due to insufficient SNR, as in the study of Gorga *et al.* (2003) where 44.2% of the I/O functions did not comply with the SNR criterion, 90% of which were related to behavioral thresholds exceeding 30 dB HL. Therefore, such hearing-loss cases would not contribute to the threshold-estimation error.

Moreover, the present study utilized optimized primary-tone levels to maximize the DPOAE amplitude by accounting for the different compressional behavior of the stimulus traveling waves at the *f*_2_-tonotopic place on the basilar membrane [Eq. (1)]. Using optimized stimulus conditions, Johnson *et al.* (2010) obtained auditory-threshold estimates with smaller estimation errors for frequencies *f*_2_ ≤ 3 kHz (except *f*_2_ = 2 kHz) than reported by Gorga *et al.* (2003). In contrast to the primary-tone levels used in the present study, the optimized parameters used for DPOAE acquisition in Johnson *et al.* (2010) did not account for two-component interference (Johnson *et al.*, 2006a), which was shown to induce a large variability in optimal primary-tone level pairs across subjects (Zelle *et al.*, 2015a). Additionally, deformations and irregularities in the shape of the I/O functions were reduced in the present dataset by a technique called high-level correction (HLC; Sec. II E), which was primarily designed to exclude saturating DPOAEs from the computation of the regression line, but also corrected for other effects causing deviation from straight-line growth, such as two-component interference in the case of continuous stimulation. Similar to the results reported by Dalhoff *et al.* (2013), the HLC algorithm did not increase threshold-estimation accuracy, but rather decreased the number of ill-defined and rejected I/O functions. For both acquisition paradigms, disabling the HLC algorithm yielded a negligible change of $\left|\sigma_{\Delta L}\right|<0.13$ dB. In contrast, Neely *et al.* (2009) accounted for deviations from straight-line growth by using two straight lines to fit the semi-logarithmic I/O functions and found a reduction of the estimation error from 14.9 to 12.5 dB.

When relating DPOAEs to hearing status, acquisition of the behavioral threshold bears additional measurement uncertainties. In the present work, to increase the accuracy of BT estimates, three consecutive measurements at *f*_2_ and neighboring frequencies were performed using a modified form of Békésy tracking audiometry (Sec. II C). The accuracy of the BT estimates was also improved by using the same ear probe for BT and DPOAE recordings. Averaging across neighboring frequencies for each *f*_2_ eliminated the fine structure in auditory thresholds. Additionally, this procedure enabled the correction of BTs for outliers and also the computation of standard deviation as a measure of reproducibility among the three Békésy measurements. The median value of the standard deviation of all BTs across frequencies and subjects was 2.37 dB, which was larger than the average standard deviation of 1.38 dB in the study of Dalhoff *et al.* (2013) but lower than the mean value of 3.9 dB in the data of Boege and Janssen (2002), both of which implemented a similar approach to estimate BTs and also used the same ear probe for BT and DPOAE recordings. In contrast, Gorga *et al.* (2003) acquired BTs using standard pure-tone audiometry with different earphones for BT and DPOAE recordings, thereby possibly inducing additional variance when relating EDPTs to BTs.

Whereas Johnson *et al.* (2007) were not able to improve accuracy by suppressing the coherent-reflection component with a third stimulus tone, the present results show a significant improvement in the accuracy of L_EHT_ when using only the nonlinear-distortion component in the regression analyses. This improvement is in accordance with previous results of Dalhoff *et al.* (2013) and supports the approach of DPOAE-component separation by exploiting the different latencies of the components, either directly in the time domain using pulsed stimuli or by means of swept primaries in combination with LSF analysis (Long *et al.*, 2008; Abdala *et al.*, 2015) or time-frequency filtering (Moleti *et al.*, 2012). Unfortunately, to our knowledge, the swept-tone technique has not yet been used to relate EDPTs to BTs. Dalhoff *et al.* (2013) investigated I/O functions recorded from 12 normal-hearing subjects for frequencies 1.5 ≤ *f*_2_ ≤ 2.5 kHz, and reported a $\sigma_{\Delta L}$ of 4.1 dB for their pulse-stimulus paradigm but 10.4 dB for continuous stimulation. This 6-dB improvement considerably exceeds that found in the present study (1.08 dB; Table II). Even if a comparable subset of the present data is considered, namely 1.5 ≤ *f*_2_ ≤ 3 kHz, and L_BT_ < 20 dB HL, the improvement is still not as large: $\sigma_{\Delta L}$ becomes 6.77 and 5.45 dB, respectively, for continuous and short-pulse stimulation; that is, the improvement is only 1.32 dB. The larger improvement afforded by pulsed stimulation in Dalhoff *et al.* (2013) may be due to their intentionally investigating a population with pronounced fine structure, whereas in the present study, the choice of frequencies and subjects was not influenced by preceding assessment of DPOAE fine structure.

The low correlation between BTs and EDPTs at *f*_2_ = 1 and 8 kHz (Table II) resulted mainly from the limited dynamic range of the DPOAE I/O function due to lack of data at elevated thresholds, which in turn adversely affected the accuracy of the estimate of the slope a of the regression line relating BTs to EDPTs. In general, a was close to 1 for short-pulse stimulation (Table II), except for *f*_2_ = 1.5 kHz (a = 0.66 ± 0.10) and 6 kHz (a = 0.82 ± 0.13). The smaller slopes at 1.5 and 6 kHz result from underestimating the hearing loss by the EDPT values, possibly related to deviations in the I/O functions induced by errors other than two-component interference, such as SNR or stimulation parameters. The slopes for the continuous data exhibit more variation, putatively due to two-component interference. Finally, the in-ear calibration procedure of the DPOAE ear probe (Sec. II B) may have influenced the accuracy of the threshold estimate due to possible calibration errors (Siegel, 1994). However, the aforementioned comparison of the present results with data reported in the literature should not be influenced by calibration concerns since those studies also employed in-ear calibration. Although measurement errors due to calibration errors cannot be excluded, results of Rogers *et al.* (2010) indicate that, compared to in-ear calibration, forward-pressure calibration yields only a minor improvement in accuracy when relating BTs to EDPTs.

### E. Diagnostic accuracy of EDPTs

Despite being related to the BT, the EDPT only provides a metric for the functional state of the cochlear amplifier, or in other words, only for the pre-neural component of cochlear function up to deflection of the IHC stereocilia. Inter-subject variation in the neural system with regard to action-potential generation, neural transmission, and other possible sources of interference must, by definition, influence the correlation between BTs and EDPTs. The significance of these influences can be gauged with the aid of a simple model presented by Dalhoff and Gummer (2011) to estimate the diagnostic accuracy of EDPTs. The model relates the standard deviation of the hearing thresholds estimated by EDPT, $\sigma_{\Delta L}$, to other sources of error by

$${\left(\sigma_{\Delta L}\right)}^{2}={\left(\sigma_{EDPT}\right)}^{2}+{\left(\sigma_{BT}\right)}^{2}+{\left(\sigma_{CA\equiv EDPT}\right)}^{2}+{\left(\sigma_{IHC+}\right)}^{2},$$ (8)

where $\sigma_{EDPT}$ and $\sigma_{BT}$ are the standard deviations of the estimates of EDPT and BT, respectively. As discussed above, they may be due to technical noise associated with single DPOAE and BT measurements, and in the case of EDPT may also contain contributions from sources which cause deviation from the idealized linear semi-logarithmic DPOAE I/O function. The term $\sigma_{CA\equiv EDPT}$ represents uncertainty in the assumption that the relation between the EDPT and the cochlear amplifier gain is the same for all subjects. Finally, the term $\sigma_{IHC+}$ represents the uncertainty in the assumption that the IHC and neural pathways are functioning normally. In general, the model is based on the assumption that all sources of error are statistically independent (for details, see Dalhoff and Gummer, 2011; Dalhoff *et al.*, 2013).

The median value ${\tilde{\sigma }}_{EDPT}$ of the population data given in Table I provides an estimate of the error of the individual regression procedures, $\sigma_{EDPT}$ = 1.54 dB (with short-pulse DPOAE and high-level correction), while the error for the behavioral threshold can be estimated by dividing the median standard deviation of the three runs, ${\tilde{\sigma }}_{BT}=$ 2.37 dB, by $\sqrt{3}$, giving $\sigma_{BT}$ = 1.37 dB. Then, the sum of the variances of the two remaining sources of error, ${\left(\sigma_{CA\equiv EDPT}\right)}^{2}+{\left(\sigma_{IHC+}\right)}^{2}$, is 38.27 dB^2^ for $\sigma_{\Delta L}$ = 6.52 dB, the standard deviation of ΔL over all frequencies for the short-pulse DPOAE data [Fig. 6(A) and Table II]. In absence of additional information, it is simply assumed that half of this variance derives from the IHC-neural source, i.e., $\sigma_{IHC+}=$ 4.37 dB. Then, an estimate of the diagnostic accuracy of cochlear amplifier function based on short-pulse DPOAE data is $\sigma_{spDP}=\sqrt{\sigma_{\Delta L}^{2}-\sigma_{\text{IHC}+}^{2}-\sigma_{\text{BT}}^{2}}=$ 4.64 dB. Since the continuous DPOAEs were recorded within the same subjects, applying $\sigma_{IHC+}$ = 4.37 dB to the continuous DPOAE data [$\sigma_{\Delta L}$ = 7.60 dB; Fig. 6(B) and Table II] yields $\sigma_{cDP}$ = 6.07 dB for the diagnostic accuracy of cochlear amplifier function using continuous stimulation. The essential step in this analysis is the (arbitrary) partitioning of the variances ${\left(\sigma_{CA\equiv EDPT}\right)}^{2}$ and ${\left(\sigma_{IHC+}\right)}^{2}$. However, the estimate of diagnostic accuracy does not critically depend on this step because ${\left(\sigma_{BT}\right)}^{2}$ and ${\left(\sigma_{EDPT}\right)}^{2}$ are relatively small compared with ${\left(\sigma_{\Delta L}\right)}^{2}$.

In summary, this analysis leads to two conclusions. First, for short-pulse DPOAEs, the error associated with diagnosing the state of the cochlear amplifier with this method has a standard deviation below 5 dB. Second, there is no evidence for an increase in the variance of the data with increasing hearing loss (cf. Fig. 6). Thus, for the interindividual variations in IHC and neural pathway functions, as given by $\sigma_{IHC+}$, a value below 5 dB appears to be a reasonable estimate, at least for the range of hearing thresholds investigated here. Nevertheless, a deviation from the reported variance might occur in studies with a larger portion of hearing-impaired subjects.

### F. Implications for clinical applications

The present data suggest that for a population with normal hearing or mild-to-moderate hearing loss, short-pulse DPOAE I/O functions enable accurate estimation of behavioral thresholds, not only for the pooled data but also for individual subjects. In the case of continuous stimulation, interference of the DPOAE components leads to significantly larger errors in the individual objective audiogram. The present work provides only a limited statement about the measurement time of short-pulse acquisition necessary in clinical routine, because identical stimulus levels were used for both the normal-hearing and the hearing-impaired group. Furthermore, the 10-dB SNR criterion for the short-pulse multi-frequency acquisition was restricted to the DPOAE with the lowest SNR within each multi-frequency acquisition sequence, causing averaging times for the remaining DPOAEs in the same sequence to be longer than necessary. On average, the measurement time to obtain threshold estimates for all eight frequencies was 16.45 ± 1.65 min and 6.85 ± 2.76 min per subject for short-pulse and continuous stimulation, respectively. These measurement times include the acquisition of eleven DPOAEs per I/O function. In the case of normal-hearing subjects, this procedure leads to oversampling of the I/O function, while for hearing-impaired subjects a large number of the L_2_ levels cannot evoke a DPOAE with suitable SNR. By extending the acquisition software to enable the selection of stimulus levels adaptively according to the SNR of the acquired DPOAEs, it should be possible to reduce the acquisition time to well below 5 min for short-pulse stimulation, feasible for daily clinical routine.

## V. CONCLUSIONS

Both DPOAE acquisition paradigms, incorporating either short-pulse stimuli or continuous primary tones, yield estimates of behavioral thresholds with high accuracy, supporting the use of frequency-specific stimulus levels and the high-level correction of semi-logarithmic I/O functions for deviations from the expected linear shape. Onset decomposition successfully extracts the nonlinear-distortion component from short-pulse DPOAE recordings. Utilizing I/O functions solely based on the extracted nonlinear-distortion components significantly improves auditory-threshold estimation for normal-hearing subjects and patients with mild-to-moderate hearing loss induced by an impaired cochlear amplifier. The high correlation of the EDPTs with behavioral thresholds demonstrates that individual audiograms representing the state of the hearing path up to the IHC stereocilia can be acquired with high reliability.

## Footnotes

^1^ See supplementary material at http://dx.doi.org/10.1121/1.4982923 E-JASMAN-141-023705 for PBF-decomposition results used to identify the interference types of the I/O functions presented in Fig. 5, with detailed fit parameters given. The estimated hearing thresholds for each subject in Fig. 5 are illustrated, in a similar vein as Fig. 9 in the manuscript. Also shown are frequency-specific scatterplots of BT as function of EDPT associated with the results shown in Table II.
